# Effects of prenatal fish oil supplementation on the development and performance of female kids after weaning

**DOI:** 10.1371/journal.pone.0310220

**Published:** 2024-09-11

**Authors:** Ibrahim Erez, Ugur Serbester

**Affiliations:** Faculty of Agriculture, Department of Animal Science, Çukurova University, Adana, Turkey; Tokat Gaziosmanpaşa University: Tokat Gaziosmanpasa Universitesi, TÜRKIYE

## Abstract

This study was performed to determine the influence of fish oil, an omega-3 fatty acids source, supplemented to diets of goats throughout all stages of gestation on the growth and milk production of weaned female kids. Eighty German Fawn (75%) x Hair (25%) crossbred goats were randomly assigned to treatment (fish oil, FiO group) and control (Rumen protected fat, RPF group) groups during the first half of pregnancy. Subsequently, the FiO group was further allocated into FiO-FiO and FiO-RPF subgroups and RPF group was further divided into RPF-FiO and RPF-RPF subgroups containing 20 goats in each during the second half of pregnancy. The growth and feed intake of 41 female kids (aged 75.1 ± 6.73 days, with a mean live weight of 11.6 ± 3.00 kg) were recorded for a 98 day post-weaning, In the continuation of the study, live weight changes, milk yield and composition of young female goats from mating to the second month of lactation and the growth of female kids until weaning were studied for a total of 210 days. Maternal nutrition slightly influenced the live weight gain of female kids over a 98-day investigation period (p = 0.070). When growth performance was considered, a higher feed conversion efficiency of female offspring was determined in RPF-FiO (5.52) treatment group compare to female kids in other treatment groups (p = 0.086). However, the maternal feeding system significantly affected live weight in the RPF-FiO treatment group during the mating period (P = 0.054). Concerning the feed intake, maternal nutrition significantly affected the feed intake of female kids (p < 0.01) with the highest feed consumption in the FiO-RPF group. The findings of this study have shown that fish oil enriched diet given to goats during gestation improved daily live weight changes and total live weight gain of female kids despite the initial disadvantage after weaning. At mating time, the live weight of young female goats in the RPF-FiO treatment group, which exhibited the highest feed conversion ratio during the 98-day study, was higher than the remaining treatment groups. Maternal nutrition had no effect on milk yield or milk components in young goats during lactation. Young female goats born to dams in the FiO-RPF group showed better performance than the other groups regarding live weight performance of their offspring on 56^th^ day postpartum.

## Introduction

Gestational factors can have effects that can lead to changes in production performance such as offspring development, reproduction and milk yield [1–3]. From the embryonic period to birth, there may be critical periods of increased tissue growth in the development of the offspring. [4]. Prenatal nutrition plays a significant role in placental-fetal development, and has a long-lasting impact on the reproductive function, mammary gland, and overall performance of offspring [5,6]. The nutrient and hormonal environment of the developing and growing fetus during these critical periods can influence expression in the fetal genome, with lifelong consequences [7]. It is known that prenatal feeding can alter epigenetic status and affect gene expression in the fetal genome [8]. Maternal feeding has been shown to influence offspring’s first and second lactation milk production as well as mammary gland development during the prenatal period [9,10]. Many fetal hormones are regulated by nutrition and this includes the IGFs and their binding proteins and insulin [11]. Besides, peroxisome proliferator-activated receptors (PPAR) belong to the family of steroid receptors and are fatty acid- activated nuclear transcription factors which are critical regulators of development and physiological function of the feto-placental unit [12]. Fatty acids, which are active membrane constituents, play crucial roles in cellular, tissue function development and signal transduction [13,14]. Polyunsaturated fatty acids (PUFAs) supplementation during pregnancy influences the fatty acid (FA) composition of some of the organs which potentially influence the neonatal fat and glucose metabolism [15].

The effects of lipids added into feed on postnatal growth performance [16], development of the reproductive system [17], milk production [18] and composition [18,19] have been investigated in farm animals.

Omega 3 fatty acids are reported to be incorporated into nerve membranes, increase protein expression at synapses, stabilize synapse regions and do this by modulating transcription factors such as PPARs [20]. Omega-3 PUFA, particularly, eicosapentaenoic acid (EPA; C20:5 n-3) and docosahexaenoic acid (DHA; C22:6 n-3), which are abundantly available in Salmon fish oil, offer a novel approach for modulating livestock metabolism [21,22]. It is thought that fish oil, which is a source of omega 3 fatty acids, can meet the increased long-chain fatty acid requirements during pregnancy and the changes that these fatty acids will cause in maternal and fetal tissues may significantly change the performance of the offspring in multiparous goats.

In the literature, there are insufficient studies on the critical effects of maternal feeding and omega-3 fatty acids on the periods of pregnancy and the adult performance of the offspring. Previous studies investigating the impact with n-3 PUFA added to dam rations for the development of their offspring have predominantly been performed in cattle and sheep for a limited timeframe, specifically during the prenatal and postpartum phases, and up to the weaning period [23,24]. However, very limited studies were carried out in goats [25]. To the best of the authors’ know, no study has explored the influence of fish oil that was supplemented to the maternal diet of goats on the development and performance of the female kids after weaning. Thus, we hypothesize that supplementing the maternal ration with fish oil throughout all stages of gestation in goats may modify the growth and milk production of female offspring. The fact that the present study aims to investigate the "intergenerational effect" of omega-3 fatty acids is one of the most important aspects of the study and it is evaluated that it will make important contributions to fill the gap in this field.

## Material and methods

### Animals, management and study design

In this study, all animals were managed and cared for according to University of Cukurova Institutional Animal Ethics Committee (Protocol number: 2–4, 2015). 80 German Fawn (75%) x Hair (25%) crossbred goats with a live weight of 47.9±3.97 kg (mean±standard deviation), aged between 2–5 years, were synchronised in heat and mating protocol was applied. The gestation period of goats can be divided into two distinct stages. The initial stage, termed the "first phase," extends from the moment of mating until the 75th day of pregnancy. The second phase encompasses the period from the 76^th^ day of gestation to parturition.

Initially, the does were randomly allocated into two groups consisting of 40 animals in each. The first group was fed fish oil during the first half of gestation (FiO group) and the second group (control) was provided with rumen protected fat (RPF group). Each group was then randomly assigned into two equal subgroups. The goats in FO group, were fed either fish oil [FiO-FiO group (n = 20)] or fish oil and protected fat [FiO-RPF group (n = 20)] in the second half of gestation. The goats in RPF group, after being divided into two subgroups, were fed similarly to the second half of the pregnancy of the FiO group [RPF-FiO group (n = 20) and RPF-RPF group (n = 20)]. After weaning, 41, 82.8-day old and weighing 11.97 kg female kids were included in the study based on the nutrition program outlined above (FiO-FiO group; n = 11, FiO-RPF group; n = 5, RPF-FiO group; n = 17, and RPF-RPF group; n = 8). Ingredients of the goat ration including saturated fat or fish oil were presented in Table 1. Nutrient requirements of goats during this period were taken from NRC [26]. Total mixed feeding (TMR) system will be applied to meet this nutrient requirement. The roughage:mixed feed ratio in the total mixture is 70:30. Shredded alfalfa hay, corn silage and wheat straw were used as roughage. The formulation of mixed feeds is made in a special feed factory. During the production phase, saturated fat in fractionated form was used in the Saturated Fat Group compound feed, and 8% from fish oil origin Salmon liquid oil source, which is rich in omega-3 fatty acids, was used in the Fish Oil group compound feed. This level was realized as 2.4% in TMR (30% x 8% = 2.4% since the mixed feed ratio in TMR is 30%). In studies using sheep and goats, the amount of fish oil consumed is 20–50 g/day [27–30]. The dry matter consumption of the project material goats was approximately 1.5 kg/day and they consumed 0.45 kg/day of mixed feed within the TMR (roughage:mixed feed ratio of 70:30). Under these conditions, approximately 36 g/day was consumed from the oil source of fish oil origin. On the other hand, considering that fish oil contains an average of 20% omega-3 fatty acid group, 7 g/day is provided from omega-3 fatty acid group. Total omega-3 fatty acid consumption was approximately 8–10 g/day, with 1–2 g/day provided by the basal diet [28,31]. Another important point is that by regulating the fat levels similarly in the groups, the mixed feed raw material composition and starch levels are similar, thus ensuring a healthier isocaloric and isonitrogenic composition.

**Table 1:** Compositions of rumen protected fat and fish oil used in the study (g/100 g oil).

Fattyacids (%)	Rumen protected fat^1^	Fish oil^2^
Myristic acid C14:0	1.18	5.02
Palmitic acid C16:0	62.11	19.85
Palmitoleic acid C16:1	-	5.73
Stearic acid C18:0	29.83	4.57
Oleic acid C18:1	6.90	17.49
Linoleic acid C18:2	**-**	1.81
Linolenic acid C18:3	-	0.88
Arachidonic acid C20:4	-	0.08
Eicosapentaenoic acid C20:5	**-**	9.75
Docosahexaenoic acid C22:6	**-**	26.2

^1^ Palm rumen protected fat (as provided by the manufacturer; Lactofat R100, Farmann, Germany).

^2^ Liquid fish oil produced from salmon (as provided by the manufacturer; Egevizyon, Turkey.

Each group of kids was housed in a 6 m x 12 m (width x length) enclosure. A three weeks adaptation period was allowed to the kids prior to treatment. Live weights of female kids were recorded as the live weights per trial on day 14 after weaning. During 57-day weaning period, kids were fed alfalfa as coarse fodder and offspring starter feed as concentrate feed *ad libitum*. Female kids were fed a total mixed ration (TMR) of concentrated feed and alfalfa hay for six months during the growth performance period. The proportion of concentrates to roughage in the total mixed ration was 85:15. Due to the fact that environmental factors are increasing day by day and epigenetic effects can be affected [32], female kids were fed high level concentrate feed (85:15) in the early period and their performances were evaluated. The kids were fed twice a day. The feeds were offered twice a day at 8.00 am and 16.00 pm as TMR. Tables 2 and 3 present the concentrated feed and TMR used including ingredients and nutritional descriptions. The kids were provided clean water *ad libitum*. Female kids were provided TMR in group enclaves on the farm after the age of six months. Four measurements of live weight were taken at 2-week intervals after birth. Until the mating season, their development was determined solely by monthly live weight measurements. Milk data were collected through a series of four tests administered at 2-weekly intervals, commencing in the fourth week of lactation.

**Table 2:** Ingredients and nutrient composition of concentrate used in the fattening of female kids (dry in air).

**Ingredients of feed**	**%**
Corn	41.4
Barley	11.3
Vibrotal	3.0
Soy bean meal(with 48% crude protein)	17.4
Wheat bran	7.5
Corn distillers dried grains with soluble (DDGS)	15.1
Fractionated oil	1.5
Salt	0.8
Vitamin-mineral mixture^1^	0.1
Sodium bicarbonate	0.8
Limestone	1.1
**Nutrient composition**	**%**
Dry matter	89.53
Crude protein	18.30
Acid Detergent Fiber	6.64
Neutral Detergent Fiber	20.01
Crude fat	3.83
Crude ash	6.90

^1^Provided per kilogram of vitamin-mineral mixture: 15.000.000 IU vitamin A, 3.000.000 IU vitamin D3, 30.000 mg Vitamin E, 150.000 mg Niacin, 10.000 mg Cu, 800 mg I, 150 mg Co, 150 mg Se, 50.000 mg Mn, 50.000 mg Fe, 50.000 mg Zn, 6.800 mg organic Mn, 1.400 mg organic Cu, 6.800 mg organic Zn, 6.800 mg organic Fe, 50 mg organic Se.

**Table 3:** Ingredients and nutrient composition of total mixed ration used in the experiment.

**Ingredients (**% of DM)	**%**
Corn	35.2
Barley	9.6
Vinasse (from yeast production)	2.6
Soybean meal	14.8
Wheat bran	6.4
Corn dry distiller grains	12.8
Rumen protected fat^1^	1.3
Sodium chloride	0.7
Vitamin and mineral premix^2^	0.09
Sodium bicarbonate	0.7
Limestone	0.9
Alfalfa hay	15.0
**Nutrient composition**	**%**
Dry matter (DM)	89.48
Crude protein (HP)	17.67
Acid Detergent Fiber (ADF)	10.72
Neutral Detergent Fiber (NDF)	23.17
Crude fat	3.51
Crude ash	7.25

^1^ Palm rumen protected fat (Lactofat R100, Farmann, Germany).

^2^ Provided per kilogram of vitamin-mineral mixture: 15.000.000 IU vitamin A, 3.000.000 IU vitamin D3, 30.000 mg Vitamin E, 150.000 mg Niacin, 10.000 mg Cu, 800 mg I, 150 mg Co, 150 mg Se, 50.000 mg Mn, 50.000 mg Fe, 50.000 mg Zn, 6.800 mg organic Mn, 1.400 mg organic Cu, 6.800 mg organic Zn, 6.800 mg organic Fe, 50 mg organic Se.

Individual live weights were determined before morning feeding. The weights of female kids were individually measured on a weekly basis. Individual live weight of doeling was measured from the mating and continued with monthly intervals until birth and then two-weekly intervals from birth to the end of the second month of suckling. Live weight gains was calculated by taking the difference between the animal’s current and previous weights and dividing this figure by the number of days between two measurements.

In this study, feed bags containing 45 kg of TMR comprising concentrated feed and dry alfalfa hay were used for feed consumption calculation. On day of feed consumption calculation, the feed left in the feeders, dropped around the feeder and the remaining feed in the bag was recorded. Then, sum of these values was subtracted from the initial weight of 45 kg to obtain the daily food consumption.

Data were collected throughout the study, spanning 210 days for the evaluation of breeding efficiency of young female goats from mating to the second month of lactation. For this purpose, we closely monitored alterations in total live weight, milk production and composition of the young female goats from mating until the second month of lactation. Additionally, we observed the growth and progress of the offspring until weaning.

To determine daily milk yield, the procedure involved separating kids from their mothers at 5.00 a.m., followed by milk monitoring at 05.00 p.m. Following lactation control, the kids were allowed to stay with their mothers. Kids were separated from their mothers again in the next day at 5.00 p.m., and milk control was performed at 5.00 a.m. The daily milk yield was determined by obtaining milk samples in the morning and evening. On days of milk controls, milk samples were collected in 50-ml skirted containers (Corning, England) for milk composition analysis. After heating each tube up to +40°C in a water bath, they were mixed together carefully.

Kids were kept with their mothers, with the exception of the days when the milk production of the mothers was measured.

### Chemical analysis of the diet

Roughage and mixed feed samples were analyzed for dry matter, crude protein, crude oil and crude ash according to methods outlined by AOAC [33], analyses of neutral detergent fiber (NDF) and acid detergent fiber (ADF) were analyzed by using a filter bag on a fiber analyzer (Ankom-200 Fiber Analyzer, ANKOM Technology Corp., New York, USA). According to the method indicated by Vansoest et al. [34], temperature resistant amylase and sodium sulfite were used in this technique.

### Statistical analysis

Data were compiled in spreadsheets (Excel 2016; Microsoft, Redmond, WA), and subsequently analyzed using SAS (version 9.4; SAS Institute Inc., Cary, NC). General linear mixed models with repeated measures were employed for the analyses, utilizing the MIXED procedure of SAS [35]. Least square means were estimated using the restricted maximum likelihood method. The statistical model incorporated the effects of treatment, time, and treatment × time (see S1 Appendix). The covariance structure used to analyze the repeated measures was a first-order heterogeneous autoregressive structure [35–37]. In all statistical analyses, when the p value less than 0.05 was considered as significant. When the p value were between 0.05 and 0.10 a trend was recognized as increasing or decreasing.

In tables, the least square means and pooled standard errors generated for these means were used. The pooled standard errors were calculated by multiplying the ’n’ of each group by its standard error, determining the grand total and then dividing this value by the total number of ’n’. When the p value was less than 0.05, means were compared using Tukey’s multiple comparison test.

## Results

The live weight at fourteen days after weaning was assumed to be the trial starting live weight of the kids. When comparing the groups based on this parameter, there was no significant difference (p > 0.05, Table 4, data were given in S2 Appendix). Female kids of goats that received protected fat during gestation exhibited the highest live weight (12.2 kg). In contrast, female kids of goats fed the fish oil diet during pregnancy had the lowest live weight (10.5 kg).

**Table 4:** The growth performances of female kids born to dams receiving omega-3 fatty acids in their rations at different stages of gestation.

	Maternal Nutrition (MN)^1^				SEM^2^	*P-value* ^*3*^		
	RPF-RPF	RPF-FiO	FiO-FiO	FiO-RPF		MN	Month	MN x Month
*n*	8	17	11	6				
Initial live weight (kg)	12.2	11.9	10.5	11.8	0.92	0.583	-	-
Live weight (kg)	19.3	17.6	19.1	19.3	0.63	0.113	<0.01	0.126
3. month	14.5	14.0	14.7	15.1	0.47			
4. month	17.9	16.2	17.9	17.8	0.62			
5. month	21.4	18.8	20.9	21.1	0.81			
6. month	23.5	21.4	22.9	23.4	0.79			
Change in live weight between the initiation of the study and the sixth month (kg)	11.5	8.97	11.2	11.1	0.82	0.070	-	-
Average daily gain (kg/day)	0.118	0.092	0.115	0.114	0.008	0.073		
Feed intake (g/day)	743.3	570.8	688.6	809.0	14.04	<0.01	<0.01	<0.01
3. month	583.9	370.8	475.4	568.3	29.61			
4. month	641.1	505.3	616.1	659.1	22.73			
5. month	859.2	720.9	735.0	819.4	9.81			
6. month	888.9	686.1	928.0	1190.3	21.81			
Total feed consumption during the experiment (kg)	75.9	58.2	68.7	79.2	1.49	<0.01	-	-
Feed conversion rate (feed intake / live weight gain)	6.81	6.49	6.16	7.65	0.53	0.371	0.016	0.144
3. month	6.04	4.77	4.46	5.44	0.93			
4. month	6.32	6.75	6.21	8.97	0.94			
5. month	7.46	10.1	7.36	7.75	1.85			
6. month	7.43	4.30	6.61	8.46	0.66			
Throughout trial	6.41	5.52	6.33	6.77	0.347	0.086	-	-

^1^RPF–RPF: Female kids born to dams fed a diet containing rumen protected fat throughout pregnancy; RPF–FiO: Female kids born to dams fed a diet containing rumen protected fat in the first half of gestation and fish oil in the second half; FiO–FiO: Female kids born to dams fed a diet containing fish oil throughout gestation; FO–RPF: Female kids born to dams fed a diet containing fish oil in the first half of gestation and rumen protected fat in the second half.

^2^ SEM: Pooled standard error of the means.

^3^ The initial live weight was used as covariant.

Statistically significant difference was not determined in live weights of the groups (p > 0.05) between the time of weaning and the age of six months. The live weights of female kids between the RPF-RPF, FiO-FiO, and FiO-RPF groups were highly similar during this period (19.3 kg, 19.1 kg, and 19.3 kg, respectively). However, the RPF-FiO group exhibited a lower live weight (17.6 kg). A statistical difference trend (p = 0.070) was observed between the live weights gained by the female kids from approximately 2.5 months of age, (initial age at the beginning of the experiment) and 6 months of age. Similarly, in the daily live weight gain calculated for this period, the female kids in the RPF-FiO treatment group had the lowest value, while the other groups had values close to each other (P = 0.073). The feed intake of female kids varied significantly (P < 0.01) across the RPF-RPF, FiO-FiO, and FiO-RPF groups. The study indicated that female kids from the FiO-RPF group exhibited the highest feed consumption rate (809 g/day), while those from the RPF-FiO group had the lowest feed consumption rate (570.8 g/day). Both month and Mother Feed × Month were determined to have a statistically significant effect on feed consumption (p < 0.01 for both). The total feed consumption of female kids from the start of the experiment until six months of age was also influenced by the maternal feeding method (p < 0.01). Female kids from the FiO-RPF group had the highest feed consumption, averaging 79.2 kg, while those from the RPF-FiO group had the lowest feed consumption, averaging 58.2 kg.

No significant differences were found between the groups regarding feed conversion ratio (p > 0.05).The findings of this study revealed that female kids born to dam goats in the RPF-FiO group exhibited the most optimal feed conversion ratio (5.52), while those born to goats in FiO-RPF group demonstrated the least favorable ratio (6.77) (p = 0.086).

Table 5 (data were given in S3 Appendix) presents the live weights of young female goats during the period between mating and parturition. A significant difference (p = 0.054) in live weights was detected during this period. Young female goats born to goats in the RPF-FiO treatment group gained the highest weight (51.3 kg), while those born to goats in the FiO-FiO treatment group gained the least weight (46.9 kg). However, no significant difference was observed between the groups considering daily live weight gain (p > 0.05). On average, the groups gained 12 kg of body weight from mating to parturition with an average of 88 grams of body weight gain per day.

**Table 5:** Comparison of the reproductive performance of female goats born to goats fed fish oil as a source of omega-3 fatty acids during various stages of gestation, from mating to parturition.

	Maternal Nutrition (MN)^1^				SEM^2^	*P-value* ^*3*^		
	RPF-RPF	RPF-FiO	FiO-FiO	FiO-RPF		MN	Month	MN x Month
*n*	8	16	10	6				
Live weight (kg)	48.7	51.3	46.9	47.3	1.31	0.054	<0.01	0.674
20. month	42.0	44.4	40.9	40.5	1.09			
21. month	46.3	48.7	45.2	45.2	1.17			
22. month	48.9	51.9	47.3	47.4	1.42			
23. month	51.9	54.9	49.1	50.3	1.62			
24. month	54.5	56.7	52.0	53.3	1.61			
Live weight change between the 20th and 24th months), kg]	12.0	12.5	11.3	12.5	1.12	0.846	-	-
Live weight change between the 20th and 24th months), g/day]	87.1	90.9	82.0	90.8	8.14	0.847	-	-

^1^RPF–RPF: Female kids born to dams fed a diet containing rumen protected fat throughout pregnancy; RPF–FiO: Female kids born to dams fed a diet containing rumen protected fat in the first half of gestation and fish oil in the second half; FiO–FiO: Female kids born to dams fed a diet containing fish oil throughout gestation; FO–RPF: Female kids born to dams fed a diet containing fish oil in the first half of gestation and rumen protected fat in the second half.

^2^ SEM: Pooled standard error of the means.

^3^ The live weight at six months of age (the conclusion of Trial II) was used as a covariate.

Statistically significant differences were not determined between treatment groups for duration of pregnancy, birth weight and milk production during weeks 2, 4, 6 and 8 (p > 0.05 for all variables). Both groups experienced an average weight loss of 2.56 kg from birth until the 56^th^ day of lactation. The average milk yield of treatment groups was 1.34 kg/day, and it was determined that maternal during gestation did not influence the milk yield of goats (p > 0.05, Table 6, data were given in S4 Appendix). Additionally, no statistically significant effect of maternal nutrition on milk composition was found (p > 0.05, Table 7, data were given in S5 and S6 Appendices).

**Table 6:** Comparison of the performance of female goats born to goat dams consuming fish oil as a source of omega-3 fatty acids during various stages of pregnancy, from birth to the second month of lactation.

	Maternal Nutrition (MN)^1^				SEM^2^	*P-value* ^3^		
	RPF-RPF	RPF-FiO	FiO-FiO	FiO-RPF		MN	Week	MN x Week
*n*	8	14	8	5				
Gestation length (day)	150.9	150.0	149.4	150.0	0.56	0.330	-	-
Colostrum IgG (g/L)	45.3	48.1	49.6	58.6	5.08	0.501	-	-
Live weight (kg)								
Parturition	48.4	49.9	47.4	50.3	2.30	0.794	-	-
Lactation	47.4	47.6	47.4	48.9	0.89	0.747	<0.01	0.993
2. weeks	49.2	48.9	48.7	50.0	0.89			
4. weeks	48.1	48.3	48.0	49.7	0.89			
6. weeks	46.1	46.6	46.0	47.7	1.09			
8. weeks	46.3	46.6	46.7	48.2	0.98			
Live weight change (Parturition– 56th day of lactation)	-2.89	-2.86	-2.82	-1.70	1.08	0.904	-	-
Milk yield (kg/day)3	1.31	1.42	1.38	1.25	0.08	0.570	<0.01	0.089
4. weeks	0.95	0.99	0.93	0.63				
6. weeks	1.08	1.05	1.08	0.94				
8. weeks	1.13	1.27	1.38	1.07				
10. weeks	2.09	2.37	2.14	2.36				

^1^RPF–RPF: Female kids born to dams fed a diet containing rumen protected fat throughout pregnancy; RPF–FiO: Female kids born to dams fed a diet containing rumen protected fat in the first half of gestation and fish oil in the second half; FiO–FiO: Female kids born to dams fed a diet containing fish oil throughout gestation; FO–RPF: Female kids born to dams fed a diet containing fish oil in the first half of gestation and rumen protected fat in the second half.

^2^ SEM: Pooled standard error of the means.

^3^ For gestation period and weight at calving, pregnancy type (single or twin) and birth weight were allocated as covariates; for live weights at lactation and milk yield, pregnancy type (single or twin) and birth weight were assigned as covariates.

**Table 7:** Comparative analysis of the milk composition of female goats born to goat dams consuming fish oil as a source of omega-3 fatty acids during various stages of pregnancy.

	Maternal Nutrition (MN)^1^				SEM^2^	*P-value*		
	RPf3F-RPF	RPF-FiO	FiO-FiO	FiO-RPF		MN	Week	MN x Week
*n*	8	14	8	5				
Total solids (%)	11.8	11.3	12.0	11.8	0.27	0.196	<0.01	0.247
4. weeks	12.7	11.7	12.3	12.5				
6. weeks	11.3	10.8	12.1	11.1				
8. weeks	11.0	11.3	11.6	11.0				
10. weeks	12.3	11.3	12.0	12.6				
Fat (%)	2.97	2.59	3.30	2.94	0.22	0.130	<0.01	0.552
4. weeks	3.68	3.05	3.51	3.76				
6. weeks	2.25	1.91	3.30	2.24				
8. weeks	2.50	2.61	2.99	2.17				
10. weeks	3.44	2.79	3.41	3.57				
Protein (%)	3.28	3.20	3.14	3.28	0.08	0.543	0.367	0.036
4. weeks	3.41	3.17	3.17	3.25				
6. weeks	3.26	3.25	3.17	3.20				
8. weeks	3.14	3.24	3.08	3.27				
10. weeks	3.32	3.14	3.13	3.40				
Lactose (%)	4.77	4.72	4.76	4.75	0.07	0.947	0.020	0.845
4. weeks	4.75	4.82	4.73	4.69				
6. weeks	4.90	4.79	4.80	4.84				
8. weeks	4.62	4.63	4.75	4.74				
10. weeks	4.81	4.66	4.76	4.75				
Casein (%)	2.69	2.60	2.59	2.69	0.07	0.601	0.138	0.088
4. weeks	2.79	2.59	2.61	2.67				
6. weeks	2.69	2.64	2.64	2.64				
8. weeks	2.53	2.61	2.53	2.64				
10. weeks	2.76	2.55	2.58	2.80				
Urea–N (mg/dl)	24.0	24.5	26.10	24.8	0.72	0.257	<0.01	0.766
4. weeks	30.7	30.4	33.3	30.2				
6. weeks	22.6	23.3	24.2	22.9				
8. weeks	21.9	22.5	23.9	24.6				
10. weeks	20.9	21.8	23.1	21.5				

^1^RPF–RPF: Female kids born to dams fed a diet containing rumen protected fat throughout pregnancy; RPF–FiO: Female kids born to dams fed a diet containing rumen protected fat in the first half of gestation and fish oil in the second half; FiO–FiO: Female kids born to dams fed a diet containing fish oil throughout gestation; FO–RPF: Female kids born to dams fed a diet containing fish oil in the first half of gestation and rumen protected fat in the second half.

^2^ SEM: Pooled standard error of the means.

^3^The pregnancy type (single or twin) and birth weight were assigned as variables for milk composition data.

A total of 59 offspring were born as a result of the breeding and mating of female young goats whose mothers had consumed fish oil at various stages of their pregnancies, with 31 females and 28 males. Two infants lost their lives shortly after birth, while the remaining 57 were successfully reared. In terms of birth weight, among groups was not found to be important difference (p > 0.05, Table 8, data were given in S7 Appendix). The average birth weight was 3.35 kg. On day 56 of the investigation, important difference (p < 0.05) was found among live weight of treatment groups. The FiO-RPF group had the highest value (9.20 kg), while the FiO-FiO group had the lowest value (7.25 kg).

**Table 8:** Comparison of the growth rates of offspring from female goats whose dams consumed fish oil as a source of omega-3 fatty acids during various stages of pregnancy.

	Maternal Nutrition (MN)^1^				SEM^2^	*P-value* ^3^		
	RPF-RPF	RPF-FiO	FiO-FiO	FiO-RPF		MN	Day	MN x Day
n	15	24	12	6				
Female	6	15	8	2				
Male	9	10^4^	4	5^4^				
Twin birth	7	11	5	2				
Twin rate	0.88	0.79	0.63	0.40				
Partum weight (kg)	3.39	3.18	3.34	3.47	0.11	0.226	-	-
Live weight (kg)	8.11	8.98	7.25	9.20	0.45	0.035	<0.01	0.207
14. day	6.24	6.62	5.38	6.77	0.34			
28. day	7.45	8.25	6.52	8.34	0.49			
42. day	9.08	9.97	8.12	10.5	0.52			
56. day	9.67	11.1	9.00	11.2	0.58			

^1^RPF–RPF: Female kids born to dams fed a diet containing rumen protected fat throughout pregnancy; RPF–FiO: Female kids born to dams fed a diet containing rumen protected fat in the first half of gestation and fish oil in the second half; FiO–FiO: Female kids born to dams fed a diet containing fish oil throughout gestation; FO–RPF: Female kids born to dams fed a diet containing fish oil in the first half of gestation and rumen protected fat in the second half.

^2^ SEM: Pooled standard error of the means.

^3^ Pregnancy type (single or twin) and dam’s birth weight were allocated as covariates for birth weight, while pregnancy type (single or twin) was assigned as covariate for the kids’ 14th, 28th, 42nd, and 56th day live weights.

^4^ One kid died shortly after birth.

## Discussion

Farmers of livestock are continually seeking innovative methods to enhance the health, productivity and development of their animals [36]. Feeding management of pregnant dams is crucial for food production due to its potential permanent effect on growth performance and health, offering a viable option for enhancing offspring productivity [37]. Omega-3 (PUFAs) can transmit the effects of embryonic development to adult cells through various mechanisms, including gene expression modification, cell membrane fluidity modification, receptor expression effects, and nuclear receptor activation [38–40]. Lopes et al. [41] have demonstrated that PUFAs positively affected developmental performance. In ruminants, fatty acid supplementation during gestation may affect the birth weight and development rate of progeny prior to and after weaning [42,43].

In the present study, initial live weights of female kids were close in all of the treatment groups (p>0.05) similar to the findings of Oviedo-Ojeda et al. [44] who found that pre-weaning (from birth to weaning) lamb growth (live weight and mean daily weight gain) was similar between the treatment groups of sheep fed either calcium salts enriched with monounsaturated fatty acids (MUFA) or EPA + DHA. Similarly, one study carried out by Coleman et al. [45]pregnant ewes fed with calcium salts supplemented with MUFA or EPA + DHA had no influence on body weight changes of lambs after weaning. However, in accordance with previous investigations, Mahboub et al. [42] and Carranza-Martin et al. [43] claimed that fish oil administration in the later stages of pregnancy caused greater lamb birth weight. Supplementing beef cows in late pregnancy with PUFA calcium salts enhanced the improvement of their offspring in the study of Marques et al. [46]. Nickles et al. [47] determined greater weaning live weight as well as the rate of weight gain per day in offspring fed higher concentrations of EPA and DHA during late gestation. Santos et al. [18] observed higher growth performance, birth weight and body condition in dairy cow calves fed PUFA near the end of gestation. However, in accordance with our study, Rosa-Velazquez et al. [24] found that the live weights of lambs delivered from ewes received with n-3 fatty acids at the last stages of pregnancy at 30 and 60 days were similar to the live weights of lambs in the control group. Similarly, supplementation of ewes with lipids in the final pregnancy period did not affect the growth of offspring [36]. Likewise, according to Annett et al. [28], adding fish oil supplement into the nutrition of 55 crossbred ewes during the latter stages of pregnancy had no effect on live weight gain of lambs. Our research also showed that the fish oil group, which started off with the lowest mean live weight (10.50 kg), caught up to the other groups’ live weight by the end of the 6-month growth performance trial. This effect might be caused by higher levels of amino acid transporter mRNA and protein expression in the offspring of pregnant dams exposed to fish oil as well as higher DNA methylation in areas related to the small intestine [48].

The effects of omega-3 PUFA added to diets at the time of gestation on the performance-related outcomes of progeny were examined in ruminant animal species [24]. Furthermore, most of the previous studies concentrated on newborn weight and its impact on how the offspring perform throughout the suckling stage of development. To our understanding, this study represents the first attempt to examine the effect of incorporating omega-3 oil-derived fish oil in the ration of maternal goats on the developmental performance of their kids after weaning. In addition, at weaning and 6 months of age, no difference was observed for live weights among groups (p > 0.05). A notable statistical distinction (p = 0.070) was identified in the trend level of live weights of female kids between the ages of 2.5 and 6 months, which corresponds to the recommended age for initiating the experiment. The female kids of goats fed RPF-FiO had the lowest live weights, whereas the female kids in the RPF-RPF, FiO-FiO and FiO-RPF groups had similar live weights. Similarly, female kids supplemented with RPF-FiO had the lowest daily body weight gain, whereas female kids in the RPF-RPF, FiO-FiO and FiO-RPF groups had similar daily body weight gains (p = 0.073). In accordance with our study, Garcia et al. [16] reported that prenatal supplementation with saturated fatty acids (SFA) during late pregnancy enhanced the average daily gain of calves (0.50 vs. 0.46 kg/day). But, Nickles et al. [47] found that omega-3 fatty acid supplementation throughout the final stages of pregnancy improved offspring performance. Marques et al. [46] also reported elevated average daily gain levels throughout both the growing and finishing phases of life in calves born to cows fed polyunsaturated fatty acids (PUFAs) in their final trimester. On the other hand, Coleman et al. [43] found that maternal nutrition enriched with fish oil in the latter days of pregnancy had no effect regarding the performance and metabolism of weaned lambs.

In the presented study, the quantity of feed consumed by female kids was significant (p < 0.01). Female kids born to goats in the FiO-RPF group had the highest feed intake (809 g/day), whereas female kids born to goats in the RPF-FiO group had the lowest feed consumption (570.8 g/day). However, according to Oviedo-Ojeda et al. [44], a greater dry matter intake was found in lambs born to dams fed monounsaturated fatty acid supplemented diet during the early stages of pregnancy. Garcia et al. [16], the addition of saturated fatty acids to the diet of cows during the later stages of pregnancy resulted in a notable increase in grain consumption among calves between 2 and 3 months of age. In research completed by Carranza-Martin et al. [43], the study showed that offspring born to dams supplemented with 0.39% calcium salts and palmitic fatty acids in the final 50 days of gestation had elevated dry matter intake (p < 0.01). Unlike Nickles et al. [47], who determined that when omega-3 fatty acids (EPA and DHA) intake increased in late pregnancy, lamb performance also increased with increasing dry matter intake. In our study, the total amount of feed consumed by female kids from the beginning of the experiment to 180 days of age was also impacted by maternal feeding (p < 0.01). The female kids born to goats in the FiO-RPF group consumed more feed (79.2 kg) than the female kids born to goats in the RPF-FiO group (58.2 kg). Alterations in the levels of hypothalamic neuropeptides have been associated with accelerated elevations in body weight gain in heifers during their formative years [49]. Appropriate fatty acid exposure during pregnancy may program the expression of several hypothalamic neuropeptides [50]. Saturated fatty acid sources may play a function in regulating food consumption [51]. In our study, the treatment group consisting of female kids fed fish oil as a supplement during the initial period of pregnancy and protected fat during the last period of pregnancy exhibited the greatest feed intake. Although, feed conversion ratio was similar in treatment groups (p > 0.05), the highest feed efficiency was observed in female offspring born to goats in the RPF-FiO group (5.52), whereas the lowest value was observed in female offspring born to goats in the FiO-RPF group (p = 0.086).

The differences (p = 0.054) between the treatment groups concerning the live weights of the developing young goats at the mating period at a tendency level. Young female goats born to goats in the RPF-FiO treatment group exhibited the highest live weight (51.3 kg), while young female goats born to dams in the FiO-FiO treatment group had the lowest live weight (46.9 kg) compared to the live weights of young female goats born to dams in the RPF-RPF treatment group (48.7 kg), and young female goats born to dams in the FiO-RPF treatment group (47.3 kg). In our study, it was observed that the RPF-FiO group showed the most favorable feed conversion rate during the six-month age period after weaning, concurrently the highest live weight during the mating period. Oviedo-Ojeda et al. [44] also found that the dry matter intake of lambs fed monounsaturated fatty acids was higher than that of lambs fed EPA and DHA (p < 0.01). In addition, the RPF-FiO treatment group, which had the highest live weight during the mating period, was thought to consume more dry matter than the other groups.

Addition of fish oil did not resulted in differences between groups regarding the duration of gestation, weight at parturition, and live weight during weeks 2, 4, 6, and 8 of lactation (p > 0.05). In all groups, daily milk production was 1.34 kg, and maternal feeding did not significantly affect the milk production (p > 0.05). Goats born to dams in the RPF-FiO group produced the most milk (1.42 kg/day) and lowest in goats born to dams in the FiO-RPF group (1.25 kg/day). According to a study by Garcia et al. [52], heifers born to fat-supplemented dams during late pregnancy produced an average of 9100 kg of milk (p = 0.09) versus 8415 kg (p = 0.10).

There are limited *in vivo* studies investigating the influence of different dietary regimens on mammary gland activity [53]. Kenyon and Blair [54] observed that maternal nutrition influences the growth of fetal mammary glands and milk production in subsequent generations. According to Van der Linden et al. [10], maternal nutrition influences offspring milk yield performance when an *ad libitum* or limited feeding regimen is applied to treatment groups separated into two sections based on their weight between the 21^th^ and 140^th^ days of pregnancy. Adiletta et al. [55] demonstrated that maternal *ad libitum* feeding influences fetal mammary gland development. Blair et al. [9] determined that the net energy and fat output of milk in the lactation milk yield period of newborn females altered with the feeding level of the dam. Changes in the metabolic activities and expression of genes responsible for proliferation and differentiation in sheep mammary epithelial cells have been linked to maternal nutrition [56]. Epigenetic modifications in the udder, which influence gene regulation, have been demonstrated to be affected by dietary fatty acids like omega-3 in pigs and goats, resulting in changes in milk composition [57]. Garcia et al. [52] reported that maternal lipid supplementation in the late uterine period affected milk and milk protein production in the first lactation period of heifers positively. In the present study, although, the young female goats born to dams in the RPF-FiO group exhibited the greatest milk yield (1.42 kg/day), the average milk production of the groups was 1.34 kg/day. Determination of 1.34 kg/day average milk yield suggested that gestational feeding of the dams did not have a statistically significant impact for milk production (p > 0.05). Concentrations of milk constituents such as milk solids, fat, protein, lactose, casein and urea-N, which make up the milk composition, were not affected by the fish oil given to the dam during pregnancy (p > 0.05).

On day 56 of the study, the groups differed significantly in terms of live weight (p < 0.05). The group FiO-RPF had the greatest value for this variable (9.20 kg), while the group FiO-FiO had the lowest value (7.25 kg). The female kids of the FiO-FiO group receiving the treatment demonstrated the lowest live weight at both the weaning stage (10.5 kg) and the mating stage (44.9 kg). According to Paten et al. [56], the mother’s weight did not have an impact on the offspring’s body weight. Notably intriguing is the fact that the offspring of the FiO-RPF group dam had a higher feed intake during the growth period compared to the offspring in the other groups.

Female kids born to dams supplemented with fish oil to the maternal ration during pregnancy showed a favourable performance during the growth period. Female kids born to dams supplemented with fish oil in the maternal ration during late pregnancy showed a favourable performance until the mating period. The F2 progeny obtained from the broodstock to which fish oil was added to the maternal diet at the first stage of gestation showed higher performance than the other treatment groups in terms of live weight at the 56th day after birth. The performance of female kids born to dams fed fish oil to the maternal ration at different stages of gestation may positively affect the performance in the early and adult period. In order to better understand this effect, some gene expression (IGF, PPARs) analyses from samples taken from different tissues of the offspring during pregnancy and after birth will contribute. The present study was limited to performance data.

## Supporting information

S1 File (RAR)

S1 Appendix The document showing statistical analysis. (PDF)

S2 Appendix Data used in Table 4. (PDF)

S3 Appendix Data used in Table 5. (PDF)

S4 Appendix Data used in Table 6. (PDF)

S5 Appendix Data used in Table 7. (PDF)

S6 Appendix Data used in Table 7. (PDF)

S7 Appendix Data used in Table 8. (PDF)

## References

[1] Funston RN, Mulliniks JT. Developmental Programming in Livestock Production. Veterinary Clinics of North America—Food Animal Practice. 2019. doi: 10.1016/j.cvfa.2019.03.001 31103190

[2] Yao S, Lopez-Tello J, Sferruzzi-Perri AN. Developmental programming of the female reproductive system—a review. Biology of Reproduction. 2021. doi: 10.1093/biolre/ioaa232 33354727

[3] Van Eetvelde M, Opsomer G. Prenatal programming of later performance in dairy cattle. Vlaams Diergeneeskd Tijdschr. 2020;89. doi: 10.21825/VDT.V89I1.15985

[4] Lukaszewski MA, Eberlé D, Vieau D, Breton C. Nutritional manipulations in the perinatal period program adipose tissue in offspring. American Journal of Physiology—Endocrinology and Metabolism. 2013. doi: 10.1152/ajpendo.00231.2013 24045869

[5] Izquierdo V, Vedovatto M, Palmer EA, Oliveira RA, Silva HM, Vendramini JMB, et al. Frequency of maternal supplementation of energy and protein during late gestation modulates preweaning growth of their beef offspring. Transl Anim Sci. 2022;6. doi: 10.1093/tas/txac110 PMC9449678 36090697

[6] National Academies of Sciences and Medicine E. Nutrient Requirements of Beef Cattle: Eighth Revised Edition. Washington, DC: The National Academies Press; 2016. doi: 10.17226/19014 38386771

[7] Godfrey K, Robinson S. Maternal nutrition, placental growth and fetal programming. Proceedings of the Nutrition Society. 1998;57. doi: 10.1079/pns19980016 9571715

[8] Waterland RA, Jirtle RL. Early nutrition, epigenetic changes at transposons and imprinted genes, and enhanced susceptibility to adult chronic diseases. Nutrition. 2004;20. doi: 10.1016/j.nut.2003.09.011 14698016

[9] Blair HT, Jenkinson CM, Peterson SW, Kenyon PR, van der Linden DS, Davenport LC, et al. Dam and granddam feeding during pregnancy in sheep affects milk supply in offspring and reproductive performance in grand-offspring. J Anim Sci. 2010;88. doi: 10.2527/jas.2009-2523 19966171

[10] van der Linden DS, Kenyon PR, Blair HT, Lopez-Villalobos N, Jenkinson CMC, Peterson SW, et al. Effects of ewe size and nutrition on fetal mammary gland development and lactational performance of offspring at their first lactation. J Anim Sci. 2009;87. doi: 10.2527/jas.2009-2125 19684261

[11] Bloomfield FH, Harding JE. Fetal nutrition. 2nd ed. In: Thureen PJ, Hay WW, editors. Neonatal Nutrition and Metabolism. 2nd ed. Cambridge: Cambridge University Press; 2006. pp. 1–22. doi: 10.1017/CBO9780511544712.002

[12] Chawta A, Repa JJ, Evans RM, Mangelsdorf DJ. Nuclear receptors and lipid physiology: Opening the x-files. Science. 2001. doi: 10.1126/science.294.5548.1866 11729302

[13] Strandvik B. Perinatal programming by diets with essential fatty acid deficient/high saturated fatty acids or different n-6/n-3 ratios for diseases in adulthood. European Journal of Lipid Science and Technology. 2015. doi: 10.1002/ejlt.201400516

[14] Bordoni A, Di Nunzio M, Danesi F, Biagi PL. Polyunsaturated fatty acids: From diet to binding to ppars and other nuclear receptors. Genes Nutr. 2006;1. doi: 10.1007/BF02829951 18850203 PMC3454679

[15] Novak EM, Keller BO, Innis SM. Metabolic development in the liver and the implications of the n-3 fatty acid supply. Am J Physiol Gastrointest Liver Physiol. 2012;302. doi: 10.1152/ajpgi.00189.2011 22094600

[16] Garcia M, Greco LF, Favoreto MG, Marsola RS, Martins LT, Bisinotto RS, et al. Effect of supplementing fat to pregnant nonlactating cows on colostral fatty acid profile and passive immunity of the newborn calf. J Dairy Sci. 2014;97. doi: 10.3168/jds.2013-7086 24239079

[17] Zachut M, Dekel I, Lehrer H, Arieli A, Arav A, Livshitz L, et al. Effects of dietary fats differing in n-6: n-3 ratio fed to high-yielding dairy cows on fatty acid composition of ovarian compartments, follicular status, and oocyte quality. J Dairy Sci. 2010;93. doi: 10.3168/jds.2009-2167 20105525

[18] Santos JEP, Greco LF, Garcia M, Thatcher WW, Staples CR, Santos JEP. The Role of Specific Fatty Acids on Dairy Cattle Performance and Fertility. Industrial farming. 2015;6.

[19] Gomes LC, Alcalde CR, Santos GT, Feihrmann AC, Molina BSL, Grande PA, et al. Concentrate with calcium salts of fatty acids increases the concentration of polyunsaturated fatty acids in milk produced by dairy goats. Small Ruminant Research. 2015;124. doi: 10.1016/j.smallrumres.2015.01.013

[20] Hajjar T, Meng GY, Rajion MA, Vidyadaran S, Othman F, Farjam AS, et al. Omega 3 polyunsaturated fatty acid improves spatial learning and hippocampal Peroxisome Proliferator Activated Receptors (PPARα and PPARγ) gene expression in rats. BMC Neurosci. 2012;13. doi: 10.1186/1471-2202-13-109 22989138 PMC3465241

[21] Rooke JA, Sinclair AG, Edwards SA, Cordoba R, Pkiyach S, Penny PC, et al. The effect of feeding salmon oil to sows throughout pregnancy on pre-weaning mortality of piglets. Animal Science. 2001;73. doi: 10.1017/s135772980005846x

[22] Coleman DN. The effects of supplementing EPA and DHA during late gestation on ewes and lambs. 2017. Available: http://rave.ohiolink.edu/etdc/view?acc_num=osu1498824557998868.

[23] Alshdaifat MMM. Effect of fish oil supplementation during gestation on maternal and offspring performance in Awassi Sheep. PhD, Çukurova University Natural and Applied Sciences. 2017.

[24] Rosa-Velazquez M, Pinos-Rodriguez JM, Parker AJ, Relling AE. Maternal supply of a source of omega-3 fatty acids and methionine during late gestation on the offspring’s growth, metabolism, carcass characteristic, and liver’s mRNA expression in sheep. J Anim Sci. 2022;100. doi: 10.1093/jas/skac032 35137115 PMC9037365

[25] Restelli L, Marques AT, Savoini G, Invernizzi G, Carisetti M, Lecchi C, et al. Saturated or unsaturated fat supplemented maternal diets influence omental adipose tissue proteome of suckling goat-kids. Res Vet Sci. 2019;125. doi: 10.1016/j.rvsc.2017.10.009 29128114

[26] NRC. Nutrient Requirements of Small Ruminants. Sheep, Goats, Cervids and New World Camelids. National Academy Press, Washington DC. 2007;09.

[27] Cattaneo D, Dell’Orto V, Varisco G, Agazzi A, Savoini G. Enrichment in n—3 fatty acids of goat’s colostrum and milk by maternal fish oil supplementation. Small Ruminant Research. 2006;64. doi: 10.1016/j.smallrumres.2005.03.013

[28] Annett RW, Dawson LER, Edgar H, Carson AF. Effects of source and level of fish oil supplementation in late pregnancy on feed intake, colostrum production and lamb output of ewes. Anim Feed Sci Technol. 2009;154. doi: 10.1016/j.anifeedsci.2009.09.002

[29] Bronzo V, Puricelli M, Agazzi A, Invernizzi G, Ferroni M, Moroni P, et al. Effects of protected fish oil in the diet of periparturient dairy goats on phenotypic variation in blood and milk leukocytes. Animal. 2010;4. doi: 10.1017/S1751731110000522 22444697

[30] Toral PG, Rouel J, Bernard L, Chilliard Y. Interaction between fish oil and plant oils or starchy concentrates in the diet: Effects on dairy performance and milk fatty acid composition in goats. Anim Feed Sci Technol. 2014;198. doi: 10.1016/j.anifeedsci.2014.09.019

[31] Capper JL, Wilkinson RG, Mackenzie AM, Sinclair LA. The effect of fish oil supplementation of pregnant and lactating ewes on milk production and lamb performance. Animal. 2007;1. doi: 10.1017/S1751731107000067 22444754

[32] Cloud J. Why Your DNA Isn’t Your Destiny. Time. 2010.

[33] AOAC International. Official methods of analysis of AOAC International. Association of Official Analysis Chemists International. 2016.

[34] Van Soest PJ, Robertson JB, Lewis BA. Methods for Dietary Fiber, Neutral Detergent Fiber, and Nonstarch Polysaccharides in Relation to Animal Nutrition. J Dairy Sci. 1991;74. doi: 10.3168/jds.S0022-0302(91)78551-2 1660498

[35] Stroup WW, Milliken GA, Claassen EA, Wolfinger RD. SAS® for Mixed Models: Introduction and Basic Applications. Cary, NC: SAS Institute Inc; 2018.

[36] Sanders AR. The effects of fatty acid supplementation during gestation on growth and glucose metabolism in the offspring in sheep. Food, Agricultural, and Environmental Sciences Undergraduate Research Theses and Honors Research Theses, The Ohio State University Ohio Agricultural Research and Development Center. 2022.

[37] Rosa-Velazquez M, Jaborek JR, Pinos-Rodriguez JM, Relling AE. Maternal supply of fatty acids during late gestation on offspring’s growth, metabolism, and carcass characteristics in sheep. Animals. 2021;11. doi: 10.3390/ani11030719 33800817 PMC8001004

[38] Sampath H, Ntambi JM. Polyunsaturated fatty acid regulation of genes of lipid metabolism. Annual Review of Nutrition. 2005. doi: 10.1146/annurev.nutr.25.051804.101917 16011470

[39] Jump DB, Tripathy S, Depner CM. Fatty acid-regulated transcription factors in the liver. Annu Rev Nutr. 2013;33. doi: 10.1146/annurev-nutr-071812-161139 23528177 PMC3940310

[40] Hon GC, Rajagopal N, Shen Y, McCleary DF, Yue F, Dang MD, et al. Epigenetic memory at embryonic enhancers identified in DNA methylation maps from adult mouse tissues. Nat Genet. 2013;45. doi: 10.1038/ng.2746 23995138 PMC4095776

[41] Lopes CN, Scarpa AB, Cappellozza BI, Cooke RF, Vasconcelos JLM. Effects of rumen-protected polyunsaturated fatty acid supplementation on reproductive performance of Bos indicus beef cows. J Anim Sci. 2009;87. doi: 10.2527/jas.2009-2201 19684273

[42] Mahboub HDH, Ramadan SGA, Helal MAY, Aziz EAK. Effect of maternal feeding in late pregnancy on behaviour and performance of Egyptian goat and sheep and their offspring. Glob Vet. 2013;11. doi: 10.5829/idosi.gv.2013.11.2.74152

[43] Martin CC, Coleman DN, Garcia LG, Furnus CC, Relling AE. Prepartum fatty acid supplementation in sheep. III. Effect of eicosapentaenoic acid and docosahexaenoic acid during finishing on performance, hypothalamus gene expression, and muscle fatty acids composition in lambs. J Anim Sci. 2018;96. doi: 10.1093/jas/sky360 30239813 PMC6276554

[44] Oviedo-Ojeda MF, Roque-Jiménez JA, Whalin M, Lee-Rangel HA, Relling AE. Effect of supplementation with different fatty acid profile to the dam in early gestation and to the offspring on the finishing diet on offspring growth and hypothalamus mRNA expression in sheep. J Anim Sci. 2021;99. doi: 10.1093/jas/skab064 33640974 PMC8075118

[45] Coleman DN, Rivera-Acevedo KC, Relling AE. Prepartum fatty acid supplementation in sheep i. Eicosapentaenoic and docosahexaenoic acid supplementation do not modify ewe and lamb metabolic status and performance through weaning. J Anim Sci. 2018;96. doi: 10.1093/jas/skx012 29365147 PMC6140940

[46] Marques RS, Cooke RF, Rodrigues MC, Brandão AP, Schubach KM, Lippolis KD, et al. Effects of supplementing calcium salts of polyunsaturated fatty acids to late-gestating beef cows on performance and physiological responses of the offspring. J Anim Sci. 2017;95. doi: 10.2527/jas2017.1606 29293770 PMC6292277

[47] Nickles KR, Hamer L, Coleman DN, Relling AE. Supplementation with eicosapentaenoic and docosahexaenoic acids in late gestation in ewes changes adipose tissue gene expression in the ewe and growth and plasma concentration of ghrelin in the offspring. J Anim Sci. 2019;97. doi: 10.1093/jas/skz141 31073599 PMC6541830

[48] Rosa Velazquez M, Batistel F, Pinos Rodriguez JM, Relling AE. Effects of maternal dietary omega-3 polyunsaturated fatty acids and methionine during late gestation on fetal growth, DNA methylation, and mRNA relative expression of genes associated with the inflammatory response, lipid metabolism and DNA methylation in placenta and offspring’s liver in sheep. J Anim Sci Biotechnol. 2020;11. doi: 10.1186/s40104-020-00513-7 33292515 PMC7672917

[49] Alves BRC, Cardoso RC, Prezotto LD, Thorson JF, Bedenbaugh M, Sharpton SM, et al. Elevated body weight gain during the juvenile period alters neuropeptide Y-gonadotropin-releasing hormone circuitry in prepubertal heifers. Biol Reprod. 2015;92. doi: 10.1095/biolreprod.114.124636 25505201

[50] Sousa-Ferreira L, Almeida LP de, Cavadas C. Role of hypothalamic neurogenesis in feeding regulation. Trends in Endocrinology and Metabolism. 2014. doi: 10.1016/j.tem.2013.10.005 24231724

[51] Fernandes MF, Mutch DM, Leri F. The relationship between fatty acids and different depression-related brain regions, and their potential role as biomarkers of response to antidepressants. Nutrients. 2017. doi: 10.3390/nu9030298 28304335 PMC5372961

[52] Garcia M, Greco LF, Block E, Santos JEP, Thatcher WW, Staples CR. Programming effect of dietary fatty acids on performance of Holstein heifers from birth through first lactation. Anim Feed Sci Technol. 2016;222. doi: 10.1016/j.anifeedsci.2016.10.003

[53] Zhang H, Ao CJ, Khas-Erdene, Song LW, Zhang XF. Effects of different model diets on milk composition and expression of genes related to fatty acid synthesis in the mammary gland of lactating dairy goats. J Dairy Sci. 2015;98. doi: 10.3168/jds.2013-7097 25981073

[54] Kenyon PR, Blair HT. Foetal programming in sheep—Effects on production. Small Ruminant Research. 2014;118. doi: 10.1016/j.smallrumres.2013.12.021

[55] Adiletta AM. Long-term effects of size and nutrition of the pregnant ewe on mammogenesis and lactation performance of offspring and growth of the grand offspring. Master, Massey University. 2012.

[56] Paten AM. Maternal nutrition programming in the sheep: Effect on postnatal growth, mammogenesis and lactatıon in adult-ewe offspring. PhD, Massey University. 2014.

[57] Lesta A, Marín-García PJ, Llobat L. How Does Nutrition Affect the Epigenetic Changes in Dairy Cows? Animals. 2023. doi: 10.3390/ani13111883 37889793 PMC10251833

