# Supplementary material for: Effects of prenatal fish oil supplementation on the development and performance of female kids after weaning
Source: PLoS One. 2024 Sep 11;19(9):e0310220. doi: 10.1371/journal.pone.0310220 (PMC11389935; doi:10.1371/journal.pone.0310220)
Supplement: S1 Appendix — (PDF) [file pone.0310220.s002.pdf]

S1 Appendix Word document showing statistical analysis

```
data table_4_data;
input ktno trt$ barnno rep bwb pregtype bwexp2s months fi_g;
datalines;
1166  fopf  1     1     2.80  2     13.25 3.00  473.7
1526  fopf  1     1     3.25  1     12.20 3.00  473.7
1376  fopf  2     2     3.20  1     16.20 3.00  699.3
…..
………
;
proc print;
run;
title 'for AIC value';
proc glimmix data= table_4_data plots=residualpanel;
class ktno trt months bwb birthtype;
model fi_g= bwexp2s trt months trt*months/ddfm=kr2;
random months / residual subject=ktno(trt) type=arh(1);
lsmeans trt/pdiff adjust=tukey adjdfe=row lines;
lsmeans trt*months/slicediff=trt adjust=tukey adjdfe=row;
store ppp;
run;
title;
ods html style=statistical sge=on;
proc plm restore=ppp;
lsmeans trt*months/ adjust=tukey plot=meanplot;
ods exclude diffs diffplot;
run;
proc plm restore=ppp;
 effectplot interaction (sliceby=months);
 run;
 proc plm restore=ppp;
 effectplot interaction (sliceby=trt);
 run;
title;
```

```
data ierez_table5;
input ktno barnno rep trt$ bbw pregttype bw_6months months bw;
datalines;

 1166    1     1      fiorpf  2.80     2      25.27  20    42.40
 1526    1     1      fiorpf  3.25     1      23.49  20    36.90
 1376    2     2      fiorpf  3.20     1      26.23  20    47.95
….
…
;
proc print;
run;
title' breeding-prepartum for AIC value';
proc glimmix ierez_table5 plots=residualpanel;
class ktno trt months;
model bw= bw_6months trt months trt*months/ddfm=kr2;
random months / residual subject=ktno(trt) type=arh(1);
lsmeans trt/pdiff adjust=tukey adjdfe=row lines;
lsmeans trt*months/slicediff=trt adjust=tukey adjdfe=row;
store ppp;
run;
```

```
data ierez_table6;
input ktno barnno rep trt$ birthtype partum_bw time postbw;
datalines;
9260  12    3     fiofio      1     57.70 1.00  53.00
1016  7     1     rpffio      1     44.70 1.00  41.70
1026  7     1     rpffio      2     51.00 1.00  46.30
1056  9     3     rpffio      2     46.75 1.00  48.20
1066  7     1     rpffio      2     45.35 1.00  46.75

…..

…..

;
proc print;
run;
title 'for AIC value';
proc glimmix data=ierez_table6 plots=residualpanel;
class ktno trt time;
model postbw= partum_bw birthtype trt time trt*time/ddfm=kr2;
random time / residual subject=ktno(trt) type=arh(1);
lsmeans trt /adjust=tukey adjdfe=row;
lsmeans trt*time/slicediff=trt adjust=tukey adjdfe=row;
store ppp;
```

```
data ierez_table7;
input ktono barnno rep trt$ bwb bw6m bwpreparl gestper birthtype bwpartum d56bw cad igg;
datalines;
9260  12    3     fiofio      1.70  25.09 58.65 149   1      57.70 49.00 -8.70 61.36
1016  7     1     rpffio      1.75  19.38 47.20 152   1      44.70 41.20 -3.50 61.36
1026  7     1     rpffio      2.35  23.32 57.00 147   2      51.00 44.50 -6.50 80.68
…..
…..
;
proc print;
run;
ods graphics on;
ods html style=statistical sge=on;
title 'disi cepiclerin dogum-laktasyon verilerinin normal dagilima uygunlugunun test
edilmesi';
proc freq data= ierez_table7;
tables trt;
run;
proc univariate data= ierez_table7;
class trt;
var bwpartum d56bw cad igg;
run;
proc univariate normal plot data= ierez_table7;
var bwpartum d56bw cad igg;
histogram bwpartum d56bw cad igg/normal (color=blue w=4);
run;
title;
title'disi cepiclerin dogum-laktasyon verilerinin varyans homojenliginin test edilmesi';
proc glm;
class trt;
model bwpartum d56bw cad igg=trt;
means trt/hovtest=bartlett;
run;
title;
quit;
proc glimmix data= ierez_table7 plots=residualpanel;
class trt birthtype;
model bwpartum= birthtype trt/ddfm=kr2;
lsmeans trt/plots=meanplot;
lsmeans birthtype/plots=meanplot;
run;
proc glimmix data= ierez_table7 plots=residualpanel;
class trt birthtype;
model gestper= birthtype trt/ddfm=kr2;
lsmeans trt/plots=meanplot;
run;
proc glimmix data= ierez_table7 plots=residualpanel;
class trt birthtype;
model d56bw= birthtype trt /ddfm=kr2;
lsmeans trt/plots=meanplot;
run;
proc glimmix data= ierez_table7 plots=residualpanel;
class trt birthtype;
model cad= birthtype trt /ddfm=kr2;
lsmeans trt/plots=meanplot;
run;
proc glimmix data= ierez_table7 plots=residualpanel;
class trt birthtype;
model igg= birthtype trt /ddfm=kr2;
lsmeans trt/plots=meanplot;
run;
```

```
data table_7_myield_comp_data;
input ktno trt$ birthtype partum_bw week myield km fat protein lakto case ureN;
datalines;
9260  fiofio      1      57.70 1.00  0.75  15.02 6.40  3.31  4.67  2.73  36.87
1016  rpffio      1      44.70 1.00  1.40  10.71 2.14  3.29  4.39  2.54  34.76
;
proc print;
run;
title 'for AIC value';
proc glimmix data= table_7_myield_comp_data plots=residualpanel;
class ktno trt week;
model myield= partum_bw birthtype trt week trt*week/ddfm=kr2;
random week / residual subject=ktno(trt) type=arh (1);
lsmeans trt /adjust=tukey adjdfe=row;
lsmeans trt*week/slicediff=trt adjust=tukey adjdfe=row;
store ppp;
run;
title;
ods html style=statistical sge=on;
proc plm restore=ppp;
lsmeans trt*week/ adjust=tukey plot=meanplot;
ods exclude diffs diffplot;
run;
proc plm restore=ppp;
 effectplot interaction (sliceby=week);
 run;
 proc plm restore=ppp;
 effectplot interaction (sliceby=trt);
run;
```

```
data table_8_data;
input atno bwp ktno trt$ sex$ birthtype bwb;
datalines;
1876  38.10 3128  pffo  Female      2     2.20
1876  38.10 3138  pffo  Female      2     1.90
1866  42.00 2108  pffo  Female      1     2.40
1396  66.00 3178  pffo  Male  2     3.40
1396  66.00 3189  pffo  Female      2     2.80
…..
…..
;
proc print;
run;
ods graphics on;
ods html style=statistical sge=on;
title 'gausian distrubition';
proc freq data= table_8_data;
tables trt;
run;
proc univariate data= table_8_data;
class trt sex;
var bwb;
run;
proc univariate normal plot data= table_8_data;
var bwb;
histogram bwb/normal (color=blue w=4);
run;
title;
title 'variance hom';
proc glm;
class trt;
model bwb=trt;
means trt/hovtest=bartlett;
run;
title;
quit;
proc glimmix data= table_8_data plots=residualpanel;
class trt birthtype sex;
model bwb= birthtype bwp sex trt/ddfm=kr2;
lsmeans trt/plots=meanplot;
lsmeans birthtype/plots=meanplot;
lsmeans sex/plots=meanplot;
run;
```

```
data table_8_1_data;
input atno bwp ktno trt$ sex$ birthtype bwb time bw;
datalines;
1876  38.10 3128  pffo  Female      2     2.20  14.00 2.75
1876  38.10 3138  pffo  Female      2     1.90  14.00 2.10
1866  42.00 2108  pffo  Female      1     2.40  14.00 6.95
1396  66.00 3178  pffo  Male  2     3.40  14.00 .
1396  66.00 3189  pffo  Female      2     2.80  14.00 .
…..
……

;
proc print;
run;
title 'for AIC';
proc glimmix data= table_8_1_data plots=residualpanel;
class ktno trt time sex;
model bw= birthtype sex bwb trt time trt*time/ddfm=kr2;
random time / residual subject=ktno(trt) type=arh(1);
lsmeans trt /adjust=tukey adjdfe=row lines;
lsmeans trt*time/slicediff=trt adjust=tukey adjdfe=row;
store ppp;
run;
title;
ods html style=statistical sge=on;
proc plm restore=ppp;
lsmeans trt*time/ adjust=tukey plot=meanplot;
ods exclude diffs diffplot;
run;
proc plm restore=ppp;
 effectplot interaction (sliceby=time);
 run;
 proc plm restore=ppp;
 effectplot interaction (sliceby=trt);
run;
```
