# Supplementary material for: Effects of prenatal fish oil supplementation on the development and performance of female kids after weaning
Source: PLoS One. 2024 Sep 11;19(9):e0310220. doi: 10.1371/journal.pone.0310220 (PMC11389935; doi:10.1371/journal.pone.0310220)
Supplement: S2 Appendix — (PDF) [file pone.0310220.s003.pdf]

kidtagno	trt	barnno	replicate	birthweight	pregtype	months	bw	fi_g	fcr
1166	fiorpf	1	1	2.80	2	3.00	16.45	473.7	4.44
1526	fiorpf	1	1	3.25	1	3.00	16.55	473.7	3.27
1376	fiorpf	2	2	3.20	1	3.00	20.20	699.3	5.25
1696	fiorpf	2	2	3.85	2	3.00	14.75	699.3	11.34
1476	fiorpf	3	3	3.05	2	3.00	13.60	532.0	3.47
1896	fiorpf	3	3	2.60	2	3.00	10.60	532.0	4.69
1416	rpfrpf	4	1	2.35	2	3.00	17.50	652.3	5.22
1506	rpfrpf	4	2	2.00	2	3.00	16.20	652.3	5.51
1126	rpfrpf	4	3	2.95	2	3.00	19.50	652.3	4.83
1616	rpfrpf	5	1	2.85	2	3.00	11.40	413.7	
1786	rpfrpf	5	2	3.15	2	3.00	15.40	413.7	3.06
1826	rpfrpf	5	3	4.65	1	3.00	15.20	413.7	3.76
1086	rpfrpf	6	1	2.45	2	3.00	12.25	737.0	13.40
1426	rpfrpf	6	3	2.45	2	3.00	13.65	737.0	5.46
1016	rpffio	7	1	1.75	2	3.00	12.90	472.7	5.56
1026	rpffio	7	2	2.35	2	3.00	15.20	472.7	
1106	rpffio	7	3	2.15	2	3.00	18.95	472.7	2.86
1396	rpffio	7	4	3.29	2	3.00	20.05	472.7	3.64
1066	rpffio	7	5	1.10	2	3.00	10.80	472.7	
1656	rpffio	7	6	3.30	1	3.00	13.90	472.7	8.34
2106	rpffio	8	1	3.05	2	3.00	10.70	303.7	2.60
1846	rpffio	8	2	3.00	2	3.00	8.35	303.7	14.02
1876	rpffio	8	3	1.85	2	3.00	8.55	303.7	3.04
2096	rpffio	8	4	3.60	2	3.00	11.85	303.7	2.28
1146	rpffio	8	5	3.20	2	3.00	11.55	303.7	6.75
1056	rpffio	9	1	2.50	2	3.00	15.85	325.0	3.10
1196	rpffio	9	2	3.35	2	3.00	19.85	325.0	3.55
1546	rpffio	9	3	3.00	1	3.00	17.00	325.0	5.91
1096	rpffio	9	4	2.60	2	3.00	18.00	325.0	3.15
1866	rpffio	9	5	1.75	2	3.00	11.05	325.0	2.95
1666	fiofio	9	6	3.25	2	3.00	18.95	325.0	3.75
1536	fiofio	10	1	3.45	1	3.00	19.60	606.7	4.14
1216	fiofio	10	2	2.40	3	3.00	16.60	606.7	6.17
1486	fiofio	10	3	3.20	2	3.00	15.95	606.7	4.09
1456	fiofio	11	1	1.76	2	3.00	12.90	446.7	9.93
1366	fiofio	11	2	2.70	3	3.00	15.25	446.7	2.79
1316	fiofio	11	3	2.00	2	3.00	9.00	446.7	
1446	fiofio	11	4	2.10	2	3.00	13.95	446.7	3.62
1356	fiofio	12	1	2.05	3	3.00	12.50	405.3	3.38
9160	fiofio	12	2	1.95	2	3.00	9.95	405.3	5.93
9260	fiofio	12	3	1.70	2	3.00	13.35	405.3	3.29
2126	fiofio	12	4	2.60	2	3.00	10.75	405.3	3.04
1166	fiorpf	1	1	2.80	2	4.00	19.75	668.7	6.08
1526	fiorpf	1	1	3.25	1	4.00	18.00	668.7	13.83
1376	fiorpf	2	2	3.20	1	4.00	21.40	577.0	14.43
1696	fiorpf	2	2	3.85	2	4.00	18.90	577.0	4.17
1476	fiorpf	3	3	3.05	2	4.00	16.30	731.7	8.13
1896	fiorpf	3	3	2.60	2	4.00	13.75	731.7	6.97
1416	rpfrpf	4	1	2.35	2	4.00	19.50	653.7	9.81

1506	rpfrpf	4	2	2.00	2	4.00	19.05	653.7	6.88
1126	rpfrpf	4	3	2.95	2	4.00	22.75	653.7	6.03
1616	rpfrpf	5	1	2.85	2	4.00	17.10	627.3	3.30
1786	rpfrpf	5	2	3.15	2	4.00	19.65	627.3	4.43
1826	rpfrpf	5	3	4.65	1	4.00	18.50	627.3	5.70
1086	rpfrpf	6	1	2.45	2	4.00	15.80	643.0	5.43
1426	rpfrpf	6	3	2.45	2	4.00	15.95	643.0	8.39
1016	rpffio	7	1	1.75	2	4.00	14.60	505.7	8.92
1026	rpffio	7	2	2.35	2	4.00	17.10	505.7	7.98
1106	rpffio	7	3	2.15	2	4.00	22.85	505.7	3.89
1396	rpffio	7	4	3.29	2	4.00	24.85	505.7	3.16
1066	rpffio	7	5	1.10	2	4.00	9.95	505.7	
1656	rpffio	7	6	3.30	1	4.00	13.85	505.7	
2106	rpffio	8	1	3.05	2	4.00	11.70	419.3	12.58
1846	rpffio	8	2	3.00	2	4.00	11.70	419.3	3.76
1876	rpffio	8	3	1.85	2	4.00	9.40	419.3	14.80
2096	rpffio	8	4	3.60	2	4.00	14.00	419.3	5.85
1146	rpffio	8	5	3.20	2	4.00	15.50	419.3	3.18
1056	rpffio	9	1	2.50	2	4.00	18.15	576.7	7.52
1196	rpffio	9	2	3.35	2	4.00	22.90	576.7	5.67
1546	rpffio	9	3	3.00	1	4.00	20.80	576.7	4.55
1096	rpffio	9	4	2.60	2	4.00	21.70	576.7	4.68
1866	rpffio	9	5	1.75	2	4.00	10.85	576.7	
1666	fiofio	9	6	3.25	2	4.00	21.30	576.7	7.36
1536	fiofio	10	1	3.45	1	4.00	22.20	761.7	8.79
1216	fiofio	10	2	2.40	3	4.00	19.25	761.7	8.62
1486	fiofio	10	3	3.20	2	4.00	18.60	761.7	8.62
1456	fiofio	11	1	1.76	2	4.00	15.90	496.7	4.97
1366	fiofio	11	2	2.70	3	4.00	18.90	496.7	4.08
1316	fiofio	11	3	2.00	2	4.00	10.50	496.7	9.93
1446	fiofio	11	4	2.10	2	4.00	17.75	496.7	3.92
1356	fiofio	12	1	2.05	3	4.00	15.90	626.0	5.52
9160	fiofio	12	2	1.95	2	4.00	14.15	626.0	4.47
9260	fiofio	12	3	1.70	2	4.00	17.35	626.0	4.70
2126	fiofio	12	4	2.60	2	4.00	13.80	626.0	6.16
1166	fiorpf	1	1	2.80	2	5.00	23.10	820.3	7.35
1526	fiorpf	1	1	3.25	1	5.00	21.25	820.3	7.57
1376	fiorpf	2	2	3.20	1	5.00	23.85	807.0	9.88
1696	fiorpf	2	2	3.85	2	5.00	22.90	741.0	5.56
1476	fiorpf	3	3	3.05	2	5.00	20.35	861.0	6.38
1896	fiorpf	3	3	2.60	2	5.00	16.45	861.0	9.57
1416	rpfrpf	4	1	2.35	2	5.00	23.75	847.3	5.98
1506	rpfrpf	4	2	2.00	2	5.00	22.60	847.3	7.16
1126	rpfrpf	4	3	2.95	2	5.00	26.25	905.0	7.76
1616	rpfrpf	5	1	2.85	2	5.00	21.25	877.0	6.34
1786	rpfrpf	5	2	3.15	2	5.00	22.20	877.0	10.32
1826	rpfrpf	5	3	4.65	1	5.00	22.10	877.0	7.31
1086	rpfrpf	6	1	2.45	2	5.00	19.05	821.7	7.58
1426	rpfrpf	6	3	2.45	2	5.00	19.70	821.7	6.57
1016	rpffio	7	1	1.75	2	5.00	16.70	718.0	10.26

1026	rpffio	7	2	2.35	2	5.00	20.45	718.0	6.43
1106	rpffio	7	3	2.15	2	5.00	26.70	691.7	5.39
1396	rpffio	7	4	3.29	2	5.00	28.05	718.0	6.73
1066	rpffio	7	5	1.10	2	5.00	11.35	691.7	14.82
1656	rpffio	7	6	3.30	1	5.00	11.55	718.0	
2106	rpffio	8	1	3.05	2	5.00	15.50	723.3	5.71
1846	rpffio	8	2	3.00	2	5.00	13.55	723.3	11.73
1876	rpffio	8	3	1.85	2	5.00	13.45	697.0	5.16
2096	rpffio	8	4	3.60	2	5.00	16.70	723.3	8.04
1146	rpffio	8	5	3.20	2	5.00	16.00	723.3	43.40
1056	rpffio	9	1	2.50	2	5.00	20.45	730.7	9.53
1196	rpffio	9	2	3.35	2	5.00	27.15	730.7	5.16
1546	rpffio	9	3	3.00	1	5.00	23.75	730.7	7.43
1096	rpffio	9	4	2.60	2	5.00	25.05	730.7	6.54
1866	rpffio	9	5	1.75	2	5.00	14.00	730.7	6.96
1666	fiofio	9	6	3.25	2	5.00	24.55	757.0	6.99
1536	fiofio	10	1	3.45	1	5.00	26.00	780.3	6.16
1216	fiofio	10	2	2.40	3	5.00	22.80	780.3	6.59
1486	fiofio	10	3	3.20	2	5.00	21.25	780.3	8.83
1456	fiofio	11	1	1.76	2	5.00	19.40	683.7	5.86
1366	fiofio	11	2	2.70	3	5.00	21.10	683.7	9.32
1316	fiofio	11	3	2.00	2	5.00	12.75	683.7	9.12
1446	fiofio	11	4	2.10	2	5.00	20.15	683.7	8.55
1356	fiofio	12	1	2.05	3	5.00	19.20	731.7	6.65
9160	fiofio	12	2	1.95	2	5.00	17.95	772.3	6.10
9260	fiofio	12	3	1.70	2	5.00	21.15	772.3	6.10
2126	fiofio	12	4	2.60	2	5.00	16.20	731.7	9.15
1166	fiorpf	1	1	2.80	2	6.00	25.27	####	8.72
1526	fiorpf	1	1	3.25	1	6.00	23.49	####	8.45
1376	fiorpf	2	2	3.20	1	6.00	26.23	####	7.96
1696	fiorpf	2	2	3.85	2	6.00	26.23	####	5.04
1476	fiorpf	3	3	3.05	2	6.00	21.61	####	13.31
1896	fiorpf	3	3	2.60	2	6.00	18.81	####	7.11
1416	rpfrpf	4	1	2.35	2	6.00	25.30	826.7	8.00
1506	rpfrpf	4	2	2.00	2	6.00	24.03	826.7	8.66
1126	rpfrpf	4	3	2.95	2	6.00	29.06	951.3	5.07
1616	rpfrpf	5	1	2.85	2	6.00	24.57	951.3	4.30
1786	rpfrpf	5	2	3.15	2	6.00	24.31	951.3	6.78
1826	rpfrpf	5	3	4.65	1	6.00	24.49	951.3	5.96
1086	rpfrpf	6	1	2.45	2	6.00	20.78	826.7	7.19
1426	rpfrpf	6	3	2.45	2	6.00	20.67	826.7	12.80
1016	rpffio	7	1	1.75	2	6.00	19.38	681.3	3.81
1026	rpffio	7	2	2.35	2	6.00	23.32	681.3	3.56
1106	rpffio	7	3	2.15	2	6.00	29.32	692.7	3.97
1396	rpffio	7	4	3.29	2	6.00	31.03	681.3	3.44
1066	rpffio	7	5	1.10	2	6.00	13.48	683.3	4.82
1656	rpffio	7	6	3.30	1	6.00	15.44	681.3	2.62
2106	rpffio	8	1	3.05	2	6.00	17.74	681.3	4.55
1846	rpffio	8	2	3.00	2	6.00	16.39	681.3	3.60
1876	rpffio	8	3	1.85	2	6.00	16.14	692.7	3.86

2096	rpffio	8	4	3.60	2	6.00	17.92	681.3	8.39
1146	rpffio	8	5	3.20	2	6.00	18.44	681.3	4.18
1056	rpffio	9	1	2.50	2	6.00	22.29	692.7	5.64
1196	rpffio	9	2	3.35	2	6.00	29.68	692.7	4.10
1546	rpffio	9	3	3.00	1	6.00	26.25	692.7	4.16
1096	rpffio	9	4	2.60	2	6.00	27.35	692.7	4.52
1866	rpffio	9	5	1.75	2	6.00	16.31	692.7	4.51
1666	fiofio	9	6	3.25	2	6.00	28.43	681.3	2.64
1536	fiofio	10	1	3.45	1	6.00	26.63	####	
1216	fiofio	10	2	2.40	3	6.00	24.37	####	10.03
1486	fiofio	10	3	3.20	2	6.00	21.99	####	
1456	fiofio	11	1	1.76	2	6.00	22.16	826.7	4.50
1366	fiofio	11	2	2.70	3	6.00	22.58	826.7	8.41
1316	fiofio	11	3	2.00	2	6.00	15.60	826.7	4.35
1446	fiofio	11	4	2.10	2	6.00	21.39	826.7	10.02
1356	fiofio	12	1	2.05	3	6.00	21.37	826.7	5.72
9160	fiofio	12	2	1.95	2	6.00	19.78	####	8.62
9260	fiofio	12	3	1.70	2	6.00	25.09	####	3.99
2126	fiofio	12	4	2.60	2	6.00	18.36	826.7	5.73
