# Supplementary material for: Effects of prenatal fish oil supplementation on the development and performance of female kids after weaning
Source: PLoS One. 2024 Sep 11;19(9):e0310220. doi: 10.1371/journal.pone.0310220 (PMC11389935; doi:10.1371/journal.pone.0310220)
Supplement: S3 Appendix — (PDF) [file pone.0310220.s004.pdf]

kidtagno	barnno	replicate	trt	birthweight	pregtype	bwg_kg	bwg_gd
1166	1	1	fiorpf	2.80	2	17.60	127.5
1526	1	1	fiorpf	3.25	1	12.25	88.8
1376	2	2	fiorpf	3.20	1	14.24	103.2
1696	2	2	fiorpf	3.85	2	5.50	39.9
1476	3	3	fiorpf	3.05	2	11.95	86.6
1896	3	3	fiorpf	2.60	2	15.40	111.6
1416	4	1	rpfrpf	2.35	2	15.05	109.1
1506	4	2	rpfrpf	2.00	2	15.93	115.4
1126	4	3	rpfrpf	2.95	2	13.70	99.3
1616	5	1	rpfrpf	2.85	2	13.65	98.9
1786	5	2	rpfrpf	3.15	2	13.35	96.7
1826	5	3	rpfrpf	4.65	1	12.85	93.1
1086	6	1	rpfrpf	2.45	2	10.10	73.2
1426	6	3	rpfrpf	2.45	2	5.20	37.7
1016	7	1	rpffio	1.75	2	13.75	99.6
1026	7	1	rpffio	2.35	2	14.00	101.4
1106	7	1	rpffio	2.15	2	14.45	104.7
1396	7	1	rpffio	3.29	2	14.85	107.6
1066	7	1	rpffio	1.10	2	14.35	104.0
1656	7	1	rpffio	3.30	1	10.75	77.9
2106	8	2	rpffio	3.05	2	16.65	120.7
1846	8	2	rpffio	3.00	2	3.30	23.9
1876	8	2	rpffio	1.85	2	8.10	58.7
2096	8	2	rpffio	3.60	2	6.05	43.8
1056	9	3	rpffio	2.50	2	9.95	72.1
1196	9	3	rpffio	3.35	2	12.30	89.1
1546	9	3	rpffio	3.00	1	14.00	101.4
1096	9	3	rpffio	2.60	2	19.55	141.7
1866	9	3	rpffio	1.75	2	8.75	63.4
1666	9	3	rpffio	3.25	2	16.50	119.6
1536	10	1	fiofio	3.45	1	12.80	92.8
1216	10	1	fiofio	2.40	3	4.60	33.3
1486	10	3	fiofio	3.20	2	9.95	72.1
1456	11	2	fiofio	1.76	2	12.00	87.0
1366	11	2	fiofio	2.70	3	12.00	87.0
1316	11	2	fiofio	2.00	2	11.20	81.2
1446	11	3	fiofio	2.10	2	16.15	117.0
1356	12	2	fiofio	2.05	3	12.65	91.7
9260	12	3	fiofio	1.70	2	9.95	72.1
2126	12	3	fiofio	2.60	2	9.85	71.4
