# Supplementary material for: Effects of prenatal fish oil supplementation on the development and performance of female kids after weaning
Source: PLoS One. 2024 Sep 11;19(9):e0310220. doi: 10.1371/journal.pone.0310220 (PMC11389935; doi:10.1371/journal.pone.0310220)
Supplement: S4 Appendix — (PDF) [file pone.0310220.s005.pdf]

kidtagno	barnno	replicate	trt	birthtype	partum_bw	time	postpbw
9260	12	3	fiofio	1	57.70	1.00	53.00
1016	7	1	rpffio	1	44.70	1.00	41.70
1026	7	1	rpffio	2	51.00	1.00	46.30
1056	9	3	rpffio	2	46.75	1.00	48.20
1066	7	1	rpffio	2	45.35	1.00	46.75
1086	6	1	rpfrpf	2	47.25	1.00	46.30
1096	9	3	rpffio	2	63.20	1.00	60.80
1106	7	1	rpffio	2	55.25	1.00	54.55
1126	4	3	rpfrpf	2	50.80	1.00	51.20
1166	1	1	fiorpf	1	52.00	1.00	55.45
1316	11	2	fiofio	1	45.35	1.00	42.20
1356	12	2	fiofio	2	45.15	1.00	46.00
1366	11	2	fiofio	2	46.25	1.00	45.15
1376	2	2	fiorpf	2	58.70	1.00	60.05
1396	7	1	rpffio	2	66.00	1.00	
1416	4	1	rpfrpf	2	47.70	1.00	48.45
1426	6	3	rpfrpf	1	44.40	1.00	45.00
1446	11	3	fiofio	1	48.70	1.00	
1456	11	2	fiofio	2	46.60	1.00	46.20
1476	3	3	fiorpf	1	51.90	1.00	45.00
1486	10	3	fiofio	2	43.60	1.00	43.90
1506	4	2	rpfrpf	2	52.35	1.00	56.50
1526	1	1	fiorpf	2	41.15	1.00	44.60
1546	9	3	rpffio	2	53.45	1.00	50.30
1616	5	1	rpfrpf	2	53.95	1.00	51.80
1656	7	1	rpffio	1	44.10	1.00	44.30
1666	9	3	rpffio	2	64.10	1.00	65.10
1786	5	2	rpfrpf	2	51.65	1.00	51.30
1826	5	3	rpfrpf	2	47.55	1.00	44.60
1866	9	3	rpffio	1	42.00	1.00	39.50
1876	8	2	rpffio	2	38.10	1.00	38.90
1896	3	3	fiorpf	1	46.35	1.00	45.80
2096	8	2	rpffio	2	46.20	1.00	46.80
2106	8	2	rpffio	2	50.20	1.00	49.75
2126	12	3	fiofio	1	45.50	1.00	46.20
9260	12	3	fiofio	1	57.70	2.00	50.95
1016	7	1	rpffio	1	44.70	2.00	42.20
1026	7	1	rpffio	2	51.00	2.00	46.75
1056	9	3	rpffio	2	46.75	2.00	46.45
1066	7	1	rpffio	2	45.35	2.00	47.35
1086	6	1	rpfrpf	2	47.25	2.00	44.25
1096	9	3	rpffio	2	63.20	2.00	58.35
1106	7	1	rpffio	2	55.25	2.00	50.70
1126	4	3	rpfrpf	2	50.80	2.00	49.30
1166	1	1	fiorpf	1	52.00	2.00	55.15
1316	11	2	fiofio	1	45.35	2.00	42.15
1356	12	2	fiofio	2	45.15	2.00	43.25
1366	11	2	fiofio	2	46.25	2.00	45.90
1376	2	2	fiorpf	2	58.70	2.00	54.45

1396	7	1	rpffio	2	66.00	2.00	69.85
1416	4	1	rpfrpf	2	47.70	2.00	48.35
1426	6	3	rpfrpf	1	44.40	2.00	44.70
1446	11	3	fiofio	1	48.70	2.00	50.10
1456	11	2	fiofio	2	46.60	2.00	45.70
1476	3	3	fiorpf	1	51.90	2.00	48.50
1486	10	3	fiofio	2	43.60	2.00	44.00
1506	4	2	rpfrpf	2	52.35	2.00	54.55
1526	1	1	fiorpf	2	41.15	2.00	45.35
1546	9	3	rpffio	2	53.45	2.00	49.15
1616	5	1	rpfrpf	2	53.95	2.00	50.50
1656	7	1	rpffio	1	44.10	2.00	44.80
1666	9	3	rpffio	2	64.10	2.00	62.35
1786	5	2	rpfrpf	2	51.65	2.00	48.90
1826	5	3	rpfrpf	2	47.55	2.00	45.95
1866	9	3	rpffio	1	42.00	2.00	41.20
1876	8	2	rpffio	2	38.10	2.00	38.75
1896	3	3	fiorpf	1	46.35	2.00	45.95
2096	8	2	rpffio	2	46.20	2.00	47.25
2106	8	2	rpffio	2	50.20	2.00	48.90
2126	12	3	fiofio	1	45.50	2.00	45.10
9260	12	3	fiofio	1	57.70	3.00	46.40
1016	7	1	rpffio	1	44.70	3.00	40.50
1026	7	1	rpffio	2	51.00	3.00	43.20
1056	9	3	rpffio	2	46.75	3.00	45.10
1066	7	1	rpffio	2	45.35	3.00	43.90
1086	6	1	rpfrpf	2	47.25	3.00	43.10
1096	9	3	rpffio	2	63.20	3.00	55.50
1106	7	1	rpffio	2	55.25	3.00	48.80
1126	4	3	rpfrpf	2	50.80	3.00	47.20
1166	1	1	fiorpf	1	52.00	3.00	53.30
1316	11	2	fiofio	1	45.35	3.00	42.50
1356	12	2	fiofio	2	45.15	3.00	41.90
1366	11	2	fiofio	2	46.25	3.00	41.60
1376	2	2	fiorpf	2	58.70	3.00	55.50
1396	7	1	rpffio	2	66.00	3.00	69.40
1416	4	1	rpfrpf	2	47.70	3.00	48.50
1426	6	3	rpfrpf	1	44.40	3.00	44.80
1446	11	3	fiofio	1	48.70	3.00	48.10
1456	11	2	fiofio	2	46.60	3.00	45.20
1476	3	3	fiorpf	1	51.90	3.00	44.20
1486	10	3	fiofio	2	43.60	3.00	43.40
1506	4	2	rpfrpf	2	52.35	3.00	51.50
1526	1	1	fiorpf	2	41.15	3.00	42.50
1546	9	3	rpffio	2	53.45	3.00	48.50
1616	5	1	rpfrpf	2	53.95	3.00	46.20
1656	7	1	rpffio	1	44.10	3.00	44.10
1666	9	3	rpffio	2	64.10	3.00	59.20
1786	5	2	rpfrpf	2	51.65	3.00	48.40
1826	5	3	rpfrpf	2	47.55	3.00	41.20

1866	9	3	rpffio	1	42.00	3.00	40.20
1876	8	2	rpffio	2	38.10	3.00	38.00
1896	3	3	fiorpf	1	46.35	3.00	43.60
2096	8	2	rpffio	2	46.20	3.00	45.10
2106	8	2	rpffio	2	50.20	3.00	48.20
2126	12	3	fiofio	1	45.50	3.00	42.00
9260	12	3	fiofio	1	57.70	4.00	49.00
1016	7	1	rpffio	1	44.70	4.00	41.20
1026	7	1	rpffio	2	51.00	4.00	44.50
1056	9	3	rpffio	2	46.75	4.00	45.10
1066	7	1	rpffio	2	45.35	4.00	45.40
1086	6	1	rpfrpf	2	47.25	4.00	43.00
1096	9	3	rpffio	2	63.20	4.00	53.50
1106	7	1	rpffio	2	55.25	4.00	50.00
1126	4	3	rpfrpf	2	50.80	4.00	47.20
1166	1	1	fiorpf	1	52.00	4.00	52.00
1316	11	2	fiofio	1	45.35	4.00	42.23
1356	12	2	fiofio	2	45.15	4.00	43.00
1366	11	2	fiofio	2	46.25	4.00	41.10
1376	2	2	fiorpf	2	58.70	4.00	53.70
1396	7	1	rpffio	2	66.00	4.00	68.00
1416	4	1	rpfrpf	2	47.70	4.00	47.50
1426	6	3	rpfrpf	1	44.40	4.00	44.40
1446	11	3	fiofio	1	48.70	4.00	47.20
1456	11	2	fiofio	2	46.60	4.00	45.20
1476	3	3	fiorpf	1	51.90	4.00	46.80
1486	10	3	fiofio	2	43.60	4.00	44.30
1506	4	2	rpfrpf	2	52.35	4.00	51.40
1526	1	1	fiorpf	2	41.15	4.00	43.00
1546	9	3	rpffio	2	53.45	4.00	47.50
1616	5	1	rpfrpf	2	53.95	4.00	46.80
1656	7	1	rpffio	1	44.10	4.00	44.30
1666	9	3	rpffio	2	64.10	4.00	60.50
1786	5	2	rpfrpf	2	51.65	4.00	46.50
1826	5	3	rpfrpf	2	47.55	4.00	45.30
1866	9	3	rpffio	1	42.00	4.00	38.10
1876	8	2	rpffio	2	38.10	4.00	39.30
1896	3	3	fiorpf	1	46.35	4.00	46.20
2096	8	2	rpffio	2	46.20	4.00	45.60
2106	8	2	rpffio	2	50.20	4.00	46.70
2126	12	3	fiofio	1	45.50	4.00	44.30
