# Supplementary material for: Effects of prenatal fish oil supplementation on the development and performance of female kids after weaning
Source: PLoS One. 2024 Sep 11;19(9):e0310220. doi: 10.1371/journal.pone.0310220 (PMC11389935; doi:10.1371/journal.pone.0310220)
Supplement: S5 Appendix — (PDF) [file pone.0310220.s006.pdf]

kidtagno	barnno	replicate	treatment	trt	birthweight	BW_0408_6months	prepartum_lastBW	gestdur
9260	12	3	fofo	fiofio	1.70	25.09	58.65	149
1016	7	1	pffo	rpffio	1.75	19.38	47.20	152
1026	7	1	pffo	rpffio	2.35	23.32	57.00	147
1056	9	3	pffo	rpffio	2.50	22.29	54.25	145
1066	7	1	pffo	rpffio	1.10	13.48	55.25	151
1086	6	1	pfpf	rpfrpf	2.45	20.78	51.55	149
1096	9	3	pffo	rpffio	2.60	27.35	67.75	149
1106	7	1	pffo	rpffio	2.15	29.32	64.35	151
1126	4	3	pfpf	rpfrpf	2.95	29.06	59.50	149
1166	1	1	fopf	fiorpf	2.80	25.27	60.00	152
1316	11	2	fofo	fiofio	2.00	15.60	45.20	151
1356	12	2	fofo	fiofio	2.05	21.37	52.00	150
1366	11	2	fofo	fiofio	2.70	22.58	51.45	149
1376	2	2	fopf	fiorpf	3.20	26.23	62.19	149
1396	7	1	pffo	rpffio	3.29	31.03	71.40	150
1416	4	1	pfpf	rpfrpf	2.35	25.30	56.75	150
1426	6	3	pfpf	rpfrpf	2.45	20.67	46.75	152
1446	11	3	fofo	fiofio	2.10	21.39	51.20	149
1456	11	2	fofo	fiofio	1.76	22.16	55.10	146
1476	3	3	fopf	fiorpf	3.05	21.61	52.45	152
1486	10	3	fofo	fiofio	3.20	21.99	45.30	151
1506	4	2	pfpf	rpfrpf	2.00	24.03	59.18	151
1526	1	1	fopf	fiorpf	3.25	23.49	49.15	146
1546	9	3	pffo	rpffio	3.00	26.25	58.70	149
1616	5	1	pfpf	rpfrpf	2.85	24.57	60.50	149
1656	7	1	pffo	rpffio	3.30	15.44	48.85	151
1666	9	3	pffo	rpffio	3.25	28.43	71.85	150
1786	5	2	pfpf	rpfrpf	3.15	24.31	60.10	149
1826	5	3	pfpf	rpfrpf	4.65	24.49	55.60	151
1866	9	3	pffo	rpffio	1.75	16.31	42.30	152
1876	8	2	pffo	rpffio	1.85	16.14	38.00	148
1896	3	3	fopf	fiorpf	2.60	18.81	52.15	152
2096	8	2	pffo	rpffio	3.60	17.92	46.20	146
2106	8	2	pffo	rpffio	3.05	17.74	59.85	149
2126	12	3	fofo	fiofio	2.60	18.36	47.05	150

pregtype	partum_bw	56d_BW	bwc	IgG
1	57.70	49.00	-8.70	61.36
1	44.70	41.20	-3.50	61.36
2	51.00	44.50	-6.50	80.68
2	46.75	45.10	-1.65	29.55
2	45.35	45.40	0.05	31.82
2	47.25	43.00	-4.25	36.36
2	63.20	53.50	-9.70	57.95
2	55.25	50.00	-5.25	72.73
2	50.80	47.20	-3.60	70.45
1	52.00	52.00	0.00	56.82
1	45.35	42.23	-3.12	31.82
2	45.15	43.00	-2.15	59.09
2	46.25	41.10	-5.15	45.45
2	58.70	53.70	-5.00	60.23
2	66.00	68.00	2.00	27.27
2	47.70	47.50	-0.20	50.00
1	44.40	44.40	0.00	50.00
1	48.70	47.20	-1.50	27.27
2	46.60	45.20	-1.40	68.18
1	51.90	46.80	-5.10	50.00
2	43.60	44.30	0.70	53.41
2	52.35	51.40	-0.95	32.95
2	41.15	43.00	1.85	72.73
2	53.45	47.50	-5.95	34.09
2	53.95	46.80	-7.15	52.27
1	44.10	44.30	0.20	50.00
2	64.10	60.50	-3.60	56.82
2	51.65	46.50	-5.15	40.91
2	47.55	45.30	-2.25	47.73
1	42.00	38.10	-3.90	42.05
2	38.10	39.30	1.20	68.18
1	46.35	46.20	-0.15	50.00
2	46.20	45.60	-0.60	32.95
2	50.20	46.70	-3.50	52.27
1	45.50	44.30	-1.20	50.00
