# Supplementary material for: Effects of prenatal fish oil supplementation on the development and performance of female kids after weaning
Source: PLoS One. 2024 Sep 11;19(9):e0310220. doi: 10.1371/journal.pone.0310220 (PMC11389935; doi:10.1371/journal.pone.0310220)
Supplement: S6 Appendix — (PDF) [file pone.0310220.s007.pdf]

kidtagno	trt	birthtype	partum_bw	week	myield	ts	fat	pro	lak	cas	ureN
1166	fiorpf	1	52.00	1.00	0.80	17.20	8.43	3.74	4.59	3.12	35.07
9260	fiofio	1	57.70	1.00	0.75	15.02	6.40	3.31	4.67	2.73	36.87
1016	rpffio	1	44.70	1.00	1.40	10.71	2.14	3.29	4.39	2.54	34.76
1026	rpffio	2	51.00	1.00	0.90	10.10	1.42	2.98	4.67	2.35	35.06
1056	rpffio	2	46.75	1.00	0.80	11.11	2.02	3.36	4.70	2.66	37.47
1066	rpffio	2	45.35	1.00	0.60	10.22	1.32	3.07	4.83	2.47	36.64
1086	rpfrpf	2	47.25	1.00	1.10	11.32	1.99	3.39	4.98	2.76	38.47
1096	rpffio	2	63.20	1.00	1.60	12.64	3.85	3.39	4.68	2.71	33.28
1106	rpffio	2	55.25	1.00	0.80	9.94	1.82	2.66	4.51	2.07	36.54
1126	rpfrpf	2	50.80	1.00	0.40	13.46	4.65	3.53	4.27	2.69	39.50
1316	fiofio	1	45.35	1.00	0.80	11.64	3.30	3.06	4.51	2.43	37.68
1356	fiofio	2	45.15	1.00	1.00	11.29	2.31	3.32	4.69	2.64	38.69
1366	fiofio	2	46.25	1.00	0.50	13.44	5.03	3.09	4.63	2.50	38.16
1376	fiorpf	2	58.70	1.00	0.70	14.34	5.41	3.52	4.71	2.87	27.84
1396	rpffio	2	66.00	1.00	0.80	18.83	10.01	3.95	4.50	3.17	43.32
1416	rpfrpf	2	47.70	1.00	0.60	12.49	3.49	3.31	4.97	2.75	33.26
1426	rpfrpf	1	44.40	1.00	1.60	11.87	2.50	3.43	5.03	2.83	32.50
1446	fiofio	1	48.70	1.00	2.00	18.81	10.03	3.96	4.48	3.16	42.87
1456	fiofio	2	46.60	1.00	0.50	12.20	3.67	3.04	4.68	2.45	35.73
1476	fiorpf	1	51.90	1.00	0.70	11.27	2.46	3.19	4.78	2.57	37.38
1486	fiofio	2	43.60	1.00	0.60	13.33	4.77	3.19	4.74	2.61	42.50
1506	rpfrpf	2	52.35	1.00	0.40	16.42	6.44	4.81	4.32	3.71	29.63
1526	fiorpf	2	41.15	1.00	0.70	12.92	4.18	3.28	4.74	2.67	37.59
1546	rpffio	2	53.45	1.00	1.60	12.60	3.81	3.29	4.70	2.66	39.31
1616	rpfrpf	2	53.95	1.00	0.60	24.85	15.57	4.79	4.18	3.81	40.23
1656	rpffio	1	44.10	1.00	0.50	17.59	9.10	3.52	9.55	3.03	27.90
1666	rpffio	2	64.10	1.00	1.60	10.21	1.89	3.06	4.32	2.32	28.45
1786	rpfrpf	2	51.65	1.00	0.60	13.96	5.39	3.41	4.45	2.70	37.57
1826	rpfrpf	2	47.55	1.00	0.90	13.44	5.43	2.94	4.48	2.36	36.84
1866	rpffio	1	42.00	1.00	0.70	11.03	2.21	3.29	4.66	2.62	35.29
1876	rpffio	2	38.10	1.00	0.70	13.80	4.91	3.81	4.44	3.04	34.77
2096	rpffio	2	46.20	1.00	0.40	15.49	7.50	2.95	4.55	2.46	33.92
2106	rpffio	2	50.20	1.00	1.10	10.70	1.66	3.15	4.89	2.55	34.62
2126	fiofio	1	45.50	1.00	1.50	11.76	2.61	3.40	4.87	2.78	37.81
9260	fiofio	1	57.70	2.00	1.10	11.17	2.26	2.98	4.92	2.54	26.62
1016	rpffio	1	44.70	2.00	1.00	11.43	2.42	3.26	4.87	2.77	27.07
1026	rpffio	2	51.00	2.00	1.20	9.57	0.82	2.97	4.79	2.42	27.63
1056	rpffio	2	46.75	2.00	1.10	10.26	1.37	3.08	4.70	2.53	29.94
1066	rpffio	2	45.35	2.00	1.00	9.90	1.79	2.73	4.39	2.17	27.80
1086	rpfrpf	2	47.25	2.00	1.00	10.35	1.10	3.27	4.90	2.71	30.60
1096	rpffio	2	63.20	2.00	1.60	10.05	1.34	2.94	4.77	2.42	24.78
1106	rpffio	2	55.25	2.00	1.30	9.24	1.09	2.65	4.51	2.12	25.01
1126	rpfrpf	2	50.80	2.00	1.00	10.39	1.47	3.00	5.05	2.55	23.02
1166	fiorpf	1	52.00	2.00	0.40	11.40	2.60	3.35	4.65	2.74	23.74
1316	fiofio	1	45.35	2.00	0.90	10.69	1.75	3.14	4.79	2.63	32.30
1356	fiofio	2	45.15	2.00	1.10	10.29	1.33	3.08	4.84	2.56	27.56
1366	fiofio	2	46.25	2.00	0.80	10.39	1.69	2.93	4.81	2.46	25.57
1376	fiorpf	2	58.70	2.00	0.40	12.54	3.81	3.02	4.88	2.63	24.70
1396	rpffio	2	66.00	2.00	0.40	14.07	5.14	3.27	4.78	2.84	18.19

1416	rpfrpf	2	47.70	2.00	1.00	11.55	2.72	3.01	4.85	2.59	23.97
1426	rpfrpf	1	44.40	2.00	0.80	10.94	1.79	3.06	5.09	2.64	25.27
1446	fiofio	1	48.70	2.00	1.10	12.00	2.88	3.44	4.77	2.90	22.60
1456	fiofio	2	46.60	2.00	0.70	10.35	1.52	2.97	4.88	2.51	31.37
1476	fiorpf	1	51.90	2.00	0.50	10.55	1.87	3.10	4.47	2.49	25.89
1486	fiofio	2	43.60	2.00	0.40	13.05	4.40	3.05	4.71	2.65	30.14
1506	rpfrpf	2	52.35	2.00	1.50	10.60	2.09	2.71	4.84	2.32	28.58
1526	fiorpf	2	41.15	2.00	1.00	10.64	1.80	3.03	4.78	2.52	25.38
1546	rpffio	2	53.45	2.00	1.40	9.98	1.19	2.96	4.78	2.45	26.23
1616	rpfrpf	2	53.95	2.00	1.10	10.50	1.02	3.46	4.87	2.85	26.70
1656	rpffio	1	44.10	2.00	1.50	11.08	2.31	3.09	4.75	2.61	24.60
1666	rpffio	2	64.10	2.00	1.00	9.30	1.20	2.80	4.42	2.22	19.48
1786	rpfrpf	2	51.65	2.00	1.50	10.18	1.07	3.19	4.91	2.65	23.33
1826	rpfrpf	2	47.55	2.00	1.00	9.64	1.05	2.87	4.71	2.36	25.74
1866	rpffio	1	42.00	2.00	0.50	12.81	4.05	3.39	4.54	2.87	27.02
1876	rpffio	2	38.10	2.00	0.40	13.18	4.29	3.40	4.66	2.89	28.67
2096	rpffio	2	46.20	2.00	0.60	10.81	2.11	2.99	4.77	2.49	25.78
2106	rpffio	2	50.20	2.00	1.50	10.03	1.04	3.10	4.86	2.57	26.77
2126	fiofio	1	45.50	2.00	0.90	13.27	4.22	3.44	4.67	2.92	27.19
9260	fiofio	1	57.70	3.00	1.30	11.06	2.00	3.16	4.93	2.63	22.35
1016	rpffio	1	44.70	3.00	1.00	11.74	2.60	3.33	4.98	2.81	23.91
1026	rpffio	2	51.00	3.00	0.50	10.57	1.95	2.96	4.77	2.44	27.98
1056	rpffio	2	46.75	3.00	1.30	10.38	1.43	3.20	4.78	2.58	28.35
1066	rpffio	2	45.35	3.00	0.80	9.87	1.28	2.96	4.67	2.36	26.28
1086	rpfrpf	2	47.25	3.00	0.80	10.33	1.09	3.29	4.89	2.66	27.52
1096	rpffio	2	63.20	3.00	1.40	9.55	0.68	3.22	4.83	2.59	21.00
1106	rpffio	2	55.25	3.00	1.00	9.08	0.81	2.66	4.62	2.09	19.85
1126	rpfrpf	2	50.80	3.00	0.90	10.13	1.32	2.97	4.87	2.43	22.49
1166	fiorpf	1	52.00	3.00	0.80	10.73	1.41	3.62	4.89	2.95	22.42
1316	fiofio	1	45.35	3.00	0.70	17.52	9.25	3.17	4.54	2.93	20.19
1356	fiofio	2	45.15	3.00	1.00	9.74	0.93	3.13	4.71	2.49	26.32
1366	fiofio	2	46.25	3.00	1.00	10.34	1.55	3.08	4.85	2.52	25.99
1376	fiorpf	2	58.70	3.00	0.90	10.17	1.21	3.03	5.08	2.56	22.87
1396	rpffio	2	66.00	3.00	0.30	14.50	5.59	3.16	4.95	2.80	14.62
1416	rpfrpf	2	47.70	3.00	1.40	11.34	2.35	3.20	4.91	2.68	19.12
1426	rpfrpf	1	44.40	3.00	1.00	10.59	1.28	3.38	5.00	2.79	19.44
1446	fiofio	1	48.70	3.00	1.30	12.66	3.67	3.33	4.86	2.83	20.13
1456	fiofio	2	46.60	3.00	1.10	10.14	1.51	2.92	4.78	2.39	25.26
1476	fiorpf	1	51.90	3.00	1.30	9.59	1.31	2.76	4.81	2.27	20.75
1486	fiofio	2	43.60	3.00	1.30	11.05	2.02	3.20	4.92	2.65	28.99
1506	rpfrpf	2	52.35	3.00	1.60	10.97	2.40	2.82	4.88	2.37	23.37
1526	fiorpf	2	41.15	3.00	0.80	10.57	2.00	2.99	4.72	2.43	21.83
1546	rpffio	2	53.45	3.00	1.70	11.40	2.07	3.45	4.94	2.86	27.67
1616	rpfrpf	2	53.95	3.00	1.00	11.18	1.44	3.63	5.13	3.03	26.39
1656	rpffio	1	44.10	3.00	1.20	10.60	1.80	3.28	4.69	2.65	20.61
1666	rpffio	2	64.10	3.00	1.70	9.52	1.44	2.90	4.37	2.24	17.66
1786	rpfrpf	2	51.65	3.00	1.30	10.19	1.22	3.28	4.79	2.65	24.86
1826	rpfrpf	2	47.55	3.00	1.00	12.51	4.08	2.96	4.71	2.53	21.36
1866	rpffio	1	42.00	3.00	0.80	10.65	0.95	3.75	4.98	3.04	28.13
1876	rpffio	2	38.10	3.00	0.60	12.91	3.89	3.59	4.66	2.97	24.78

2096	rpffio	2	46.20	3.00	1.20	9.98	0.98	3.09	4.94	2.54	23.14
2106	rpffio	2	50.20	3.00	1.50	9.96	0.84	3.32	4.83	2.67	23.95
2126	fiofio	1	45.50	3.00	1.40	12.42	3.69	3.26	4.76	2.75	23.16
9260	fiofio	1	57.70	4.00	0.90	11.11	1.89	3.24	5.03	2.68	25.30
1016	rpffio	1	44.70	4.00	0.60	15.23	6.43	3.40	4.76	2.94	21.58
1026	rpffio	2	51.00	4.00	1.10	10.27	1.77	2.97	4.71	2.37	25.85
1056	rpffio	2	46.75	4.00	1.10	10.97	1.95	3.24	4.86	2.63	27.22
1066	rpffio	2	45.35	4.00	0.90	9.83	0.91	3.19	4.77	2.52	32.01
1086	rpfrpf	2	47.25	4.00	0.80	13.10	4.22	3.31	4.75	2.77	25.30
1096	rpffio	2	63.20	4.00	1.10	9.99	1.21	3.23	4.80	2.61	21.43
1106	rpffio	2	55.25	4.00	0.60	9.46	1.03	2.84	4.62	2.22	19.78
1126	rpfrpf	2	50.80	4.00	0.90	10.18	1.12	3.22	4.90	2.56	22.67
1166	fiorpf	1	52.00	4.00	0.80	15.28	6.34	3.77	4.55	3.16	16.19
1316	fiofio	1	45.35	4.00	1.20	11.47	2.66	3.27	4.76	2.66	26.28
1356	fiofio	2	45.15	4.00	0.70	11.08	1.92	3.50	4.81	2.78	27.92
1366	fiofio	2	46.25	4.00	0.90	11.24	2.31	3.23	4.89	2.67	27.09
1376	fiorpf	2	58.70	4.00	1.00	10.59	1.43	3.26	5.17	2.74	23.52
1396	rpffio	2	66.00	4.00	0.90	10.45	1.16	3.55	4.90	2.85	16.34
1416	rpfrpf	2	47.70	4.00	1.30	11.56	2.79	3.13	4.89	2.59	16.49
1426	rpfrpf	1	44.40	4.00	0.90	10.78	1.56	3.40	5.02	2.77	19.11
1446	fiofio	1	48.70	4.00	1.00	13.23	4.17	3.44	4.85	2.87	20.45
1456	fiofio	2	46.60	4.00	1.00	14.50	6.33	2.89	4.57	2.49	23.39
1476	fiorpf	1	51.90	4.00	1.20	11.18	2.40	3.13	4.84	2.58	28.39
1486	fiofio	2	43.60	4.00	1.30	14.40	5.72	3.20	4.73	2.74	21.93
1506	rpfrpf	2	52.35	4.00	1.50	11.83	2.99	3.05	4.99	2.56	26.41
1526	fiorpf	2	41.15	4.00	0.90	11.11	2.34	3.27	4.74	2.66	23.24
1546	rpffio	2	53.45	4.00	1.20	11.56	2.28	3.33	5.07	2.78	25.12
1616	rpfrpf	2	53.95	4.00	1.00	10.89	1.21	3.65	5.16	2.98	19.56
1656	rpffio	1	44.10	4.00	1.30	11.30	2.36	3.43	4.84	2.78	22.72
1666	rpffio	2	64.10	4.00	1.20	9.00	0.91	2.81	4.58	2.15	17.70
1786	rpfrpf	2	51.65	4.00	0.50	13.10	4.53	3.37	4.49	2.74	23.04
1826	rpfrpf	2	47.55	4.00	1.20	10.30	1.47	3.15	4.92	2.58	28.50
1866	rpffio	1	42.00	4.00	1.00	10.43	1.15	3.75	4.80	2.97	25.55
1876	rpffio	2	38.10	4.00	1.00	11.03	1.79	3.60	4.82	2.92	21.56
2096	rpffio	2	46.20	4.00	1.10	10.24	1.49	3.11	4.82	2.48	22.96
2106	rpffio	2	50.20	4.00	1.50	10.16	1.24	3.32	4.75	2.60	23.19
2126	fiofio	1	45.50	4.00	1.00	14.07	5.19	3.40	4.85	2.91	24.28
9260	fiofio	1	57.70	5.00	.	12.71	4.15	2.96	4.77	2.45	18.75
1016	rpffio	1	44.70	5.00	0.90	12.46	3.75	3.30	4.69	2.66	25.19
1026	rpffio	2	51.00	5.00	.	13.31	5.45	2.73	4.52	2.25	26.14
1056	rpffio	2	46.75	5.00	1.30	11.11	2.30	3.28	4.59	2.58	24.20
1066	rpffio	2	45.35	5.00	1.00	10.42	1.72	3.07	4.71	2.44	30.55
1086	rpfrpf	2	47.25	5.00	1.00	10.61	1.59	3.26	4.92	2.64	26.14
1096	rpffio	2	63.20	5.00	1.20	9.86	1.03	3.27	4.85	2.63	19.26
1106	rpffio	2	55.25	5.00	1.10	9.40	0.96	2.84	4.76	2.26	20.84
1126	rpfrpf	2	50.80	5.00	1.30	9.87	1.14	3.10	4.83	2.46	18.93
1166	fiorpf	1	52.00	5.00	1.10	10.70	1.53	3.67	4.78	2.90	20.12
1316	fiofio	1	45.35	5.00	0.60	10.93	2.31	3.23	4.62	2.58	27.02
1356	fiofio	2	45.15	5.00	1.00	10.81	1.51	3.35	5.06	2.71	29.26
1366	fiofio	2	46.25	5.00	1.30	11.74	3.06	3.03	4.81	2.52	18.83

1376	fiorpf	2	58.70	5.00	0.60	10.79	1.71	3.16	5.15	2.66	25.19
1396	rpffio	2	66.00	5.00	0.80	10.88	1.66	3.38	5.07	2.77	16.18
1416	rpfrpf	2	47.70	5.00	1.00	11.01	2.42	3.00	4.89	2.45	21.97
1426	rpfrpf	1	44.40	5.00	0.90	10.84	1.68	3.46	4.88	2.80	20.14
1446	fiofio	1	48.70	5.00	1.50	12.98	4.54	3.06	4.68	2.49	17.03
1456	fiofio	2	46.60	5.00	0.50	13.38	5.29	2.96	4.42	2.42	24.67
1476	fiorpf	1	51.90	5.00	0.90	11.96	3.57	3.07	4.68	2.48	17.89
1486	fiofio	2	43.60	5.00	1.60	11.26	2.31	3.21	4.96	2.67	26.32
1506	rpfrpf	2	52.35	5.00	1.40	13.42	4.93	2.91	4.87	2.49	23.76
1526	fiorpf	2	41.15	5.00	1.10	11.70	3.04	3.27	4.69	2.65	20.34
1546	rpffio	2	53.45	5.00	1.40	11.87	2.86	3.23	4.96	2.69	26.40
1616	rpfrpf	2	53.95	5.00	0.80	11.18	1.81	3.34	5.16	2.74	20.22
1656	rpffio	1	44.10	5.00	1.40	11.33	2.53	3.41	4.75	2.72	19.50
1666	rpffio	2	64.10	5.00	1.30	9.95	1.57	3.07	4.62	2.40	18.63
1786	rpfrpf	2	51.65	5.00	1.20	10.43	1.69	3.25	4.74	2.61	24.75
1826	rpfrpf	2	47.55	5.00	1.10	11.62	2.93	3.19	4.84	2.62	26.94
1866	rpffio	1	42.00	5.00	1.30	11.38	2.46	3.54	4.73	2.84	25.59
1876	rpffio	2	38.10	5.00	0.60	11.61	2.62	3.34	4.85	2.75	24.00
2096	rpffio	2	46.20	5.00	1.10	10.35	1.55	3.25	4.71	2.55	22.09
2106	rpffio	2	50.20	5.00	1.30	10.53	1.61	3.42	4.70	2.70	22.04
2126	fiofio	1	45.50	5.00	1.30	13.35	4.46	3.42	4.82	2.89	24.26
9260	fiofio	1	57.70	6.00	1.80	14.54	5.96	3.14	4.63	2.69	21.38
1016	rpffio	1	44.70	6.00	1.30	11.18	2.32	3.22	4.85	2.67	22.87
1026	rpffio	2	51.00	6.00	1.30	13.43	5.63	2.77	4.39	2.35	24.29
1056	rpffio	2	46.75	6.00	1.80	15.59	6.99	3.38	4.54	2.93	15.30
1066	rpffio	2	45.35	6.00	1.20	9.48	1.97	2.99	3.46	2.03	24.65
1086	rpfrpf	2	47.25	6.00	1.20	12.01	3.30	3.28	4.68	2.71	22.62
1096	rpffio	2	63.20	6.00	1.60	9.55	0.81	3.20	4.71	2.56	21.30
1106	rpffio	2	55.25	6.00	1.30	8.82	1.02	2.80	4.11	2.06	17.08
1126	rpfrpf	2	50.80	6.00	1.20	15.02	6.72	3.01	4.67	2.64	11.95
1166	fiorpf	1	52.00	6.00	1.30	11.15	1.75	3.85	4.70	3.08	22.74
1316	fiofio	1	45.35	6.00	0.80	12.83	4.33	2.81	4.87	2.44	35.63
1356	fiofio	2	45.15	6.00	1.70	10.20	1.49	3.03	4.81	2.47	27.62
1366	fiofio	2	46.25	6.00	1.40	10.34	1.60	3.15	4.74	2.55	26.41
1376	fiorpf	2	58.70	6.00	1.20	10.35	1.68	3.23	4.56	2.55	24.56
1396	rpffio	2	66.00	6.00	1.10	11.18	2.01	3.60	4.87	2.95	21.57
1416	rpfrpf	2	47.70	6.00	1.20	10.50	1.36	3.42	4.77	2.74	20.17
1426	rpfrpf	1	44.40	6.00	1.10	10.42	1.39	3.30	4.92	2.70	17.64
1446	fiofio	1	48.70	6.00	2.00	11.84	2.93	3.30	4.84	2.76	16.42
1456	fiofio	2	46.60	6.00	1.50	9.21	0.76	3.03	4.50	2.35	23.27
1476	fiorpf	1	51.90	6.00	1.30	10.53	1.94	3.07	4.67	2.49	26.72
1486	fiofio	2	43.60	6.00	1.80	11.22	2.51	3.07	4.79	2.55	26.42
1506	rpfrpf	2	52.35	6.00	1.80	5.07	1.21	1.37	1.93	0.37	15.84
1526	fiorpf	2	41.15	6.00	1.20	11.21	2.63	3.05	4.71	2.50	35.28
1546	rpffio	2	53.45	6.00	1.80	10.93	2.19	3.09	4.72	2.53	27.60
1616	rpfrpf	2	53.95	6.00	0.60	11.81	2.35	3.81	4.77	3.08	23.70
1656	rpffio	1	44.10	6.00	1.70	12.23	3.24	3.49	4.78	2.89	22.03
1666	rpffio	2	64.10	6.00	1.90	9.42	1.44	2.94	4.27	2.25	19.16
1786	rpfrpf	2	51.65	6.00	1.00	10.42	1.86	3.20	4.58	2.55	36.85
1826	rpfrpf	2	47.55	6.00	1.20	10.82	2.59	3.03	4.49	2.43	23.04

1866	rpffio	1	42.00	6.00	0.80	12.06	2.89	3.80	4.57	3.07	25.40
1876	rpffio	2	38.10	6.00	0.70	10.83	1.99	3.33	4.67	2.70	23.50
2096	rpffio	2	46.20	6.00	1.50	14.22	5.85	3.15	4.49	2.68	16.68
2106	rpffio	2	50.20	6.00	1.70	10.07	1.15	3.32	4.63	2.62	25.28
2126	fiofio	1	45.50	6.00	1.30	11.28	2.68	3.17	4.72	2.62	20.80
9260	fiofio	1	57.70	7.00	2.10	11.96	3.94	2.67	4.56	2.20	31.95
1016	rpffio	1	44.70	7.00	1.70	11.31	3.14	2.96	4.50	2.41	19.85
1026	rpffio	2	51.00	7.00	2.30	9.21	1.97	2.40	4.17	1.80	20.87
1056	rpffio	2	46.75	7.00	3.30	11.91	2.99	3.35	4.72	2.73	26.72
1066	rpffio	2	45.35	7.00	1.90	9.66	2.18	2.59	4.06	1.91	21.66
1086	rpfrpf	2	47.25	7.00	2.20	11.50	2.65	3.27	4.78	2.69	22.21
1096	rpffio	2	63.20	7.00	2.90	10.21	1.63	3.10	4.80	2.53	17.54
1106	rpffio	2	55.25	7.00	2.30	.	.	.	.	.	.
1126	rpfrpf	2	50.80	7.00	1.90	11.37	2.45	3.30	4.81	2.71	19.83
1166	fiorpf	1	52.00	7.00	2.00	12.34	3.03	3.92	4.70	3.16	19.94
1316	fiofio	1	45.35	7.00	2.00	11.11	2.90	3.03	4.53	2.44	19.41
1356	fiofio	2	45.15	7.00	2.40	12.01	3.24	3.39	4.66	2.72	22.28
1366	fiofio	2	46.25	7.00	2.00	12.33	3.74	3.22	4.71	2.66	21.66
1376	fiorpf	2	58.70	7.00	2.60	11.62	2.50	3.48	4.96	2.88	21.50
1396	rpffio	2	66.00	7.00	1.70	10.64	1.71	3.33	4.80	2.70	17.28
1416	rpfrpf	2	47.70	7.00	2.20	12.15	3.21	3.26	4.93	2.73	18.17
1426	rpfrpf	1	44.40	7.00	1.70	12.03	2.79	3.44	5.01	2.89	18.67
1446	fiofio	1	48.70	7.00	2.30	11.60	2.92	3.07	4.91	2.55	19.83
1456	fiofio	2	46.60	7.00	1.80	13.06	4.29	3.22	4.73	2.69	24.00
1476	fiorpf	1	51.90	7.00	2.20	11.38	3.34	2.83	4.50	2.29	19.63
1486	fiofio	2	43.60	7.00	2.50	10.82	2.01	3.17	4.81	2.58	24.93
1506	rpfrpf	2	52.35	7.00	2.50	12.98	4.36	2.98	4.86	2.55	24.37
1526	fiorpf	2	41.15	7.00	3.00	11.80	2.78	3.37	4.77	2.76	20.11
1546	rpffio	2	53.45	7.00	2.80	12.46	3.70	3.12	4.78	2.61	23.15
1616	rpfrpf	2	53.95	7.00	1.70	12.68	3.41	3.56	4.99	2.97	17.90
1656	rpffio	1	44.10	7.00	2.10	11.75	2.71	3.59	4.80	2.92	22.05
1666	rpffio	2	64.10	7.00	2.70	9.64	2.00	2.77	4.15	2.07	13.59
1786	rpfrpf	2	51.65	7.00	1.60	10.40	2.34	3.09	4.32	2.42	23.80
1826	rpfrpf	2	47.55	7.00	1.90	13.04	4.40	3.36	4.60	2.77	23.90
1866	rpffio	1	42.00	7.00	1.70	11.12	2.61	3.56	4.30	2.75	20.92
1876	rpffio	2	38.10	7.00	1.90	11.06	2.11	3.27	4.88	2.72	19.65
2096	rpffio	2	46.20	7.00	3.30	10.00	2.00	2.84	4.37	2.16	20.46
2106	rpffio	2	50.20	7.00	3.10	11.27	2.26	3.20	5.05	2.68	26.05
2126	fiofio	1	45.50	7.00	1.40	13.00	4.15	3.38	4.83	2.86	20.47
9260	fiofio	1	57.70	8.00	3.30	11.94	2.87	3.21	4.95	2.69	23.27
1016	rpffio	1	44.70	8.00	1.90	13.47	4.90	3.14	4.82	2.69	21.56
1026	rpffio	2	51.00	8.00	2.40	11.64	3.20	2.94	4.76	2.42	23.49
1056	rpffio	2	46.75	8.00	2.30	12.09	3.23	3.32	4.67	2.71	27.72
1066	rpffio	2	45.35	8.00	2.50	11.76	3.19	3.13	4.62	2.52	27.34
1086	rpfrpf	2	47.25	8.00	2.30	11.45	2.36	3.36	4.90	2.75	21.71
1096	rpffio	2	63.20	8.00	3.00	10.74	2.49	2.82	4.78	2.33	17.28
1106	rpffio	2	55.25	8.00	2.00	10.76	2.52	2.83	4.66	2.30	20.47
1126	rpfrpf	2	50.80	8.00	2.10	11.22	2.30	3.22	4.85	2.64	17.91
1166	fiorpf	1	52.00	8.00	2.10	12.82	3.53	3.81	4.78	3.14	21.84
1316	fiofio	1	45.35	8.00	1.80	11.64	3.05	3.12	4.75	2.58	23.02

1356	fiofio	2	45.15	8.00	2.00	12.85	4.23	3.26	4.68	2.68	22.68
1366	fiofio	2	46.25	8.00	2.10	12.76	4.02	3.22	4.88	2.68	22.25
1376	fiorpf	2	58.70	8.00	2.50	13.49	4.42	3.62	4.79	3.04	22.54
1396	rpffio	2	66.00	8.00	1.90	12.18	3.18	3.43	4.85	2.84	18.63
1416	rpfrpf	2	47.70	8.00	2.00	13.60	4.76	3.25	4.85	2.76	15.13
1426	rpfrpf	1	44.40	8.00	2.40	14.47	5.53	3.36	4.85	2.90	19.09
1446	fiofio	1	48.70	8.00	2.60	12.69	4.23	2.98	4.85	2.50	17.39
1456	fiofio	2	46.60	8.00	2.00	11.66	2.73	3.19	4.90	2.66	24.05
1476	fiorpf	1	51.90	8.00	2.40	12.63	4.02	3.13	4.74	2.61	22.65
1486	fiofio	2	43.60	8.00	2.00	12.29	3.71	3.14	4.64	2.58	28.36
1506	rpfrpf	2	52.35	8.00	3.00	13.45	4.75	3.09	4.84	2.67	25.63
1526	fiorpf	2	41.15	8.00	2.20	14.14	5.42	3.21	4.71	2.74	19.73
1546	rpffio	2	53.45	8.00	2.80	13.03	4.04	3.34	4.88	2.82	23.47
1616	rpfrpf	2	53.95	8.00	2.00	12.43	2.84	3.65	5.15	3.04	20.08
1656	rpffio	1	44.10	8.00	2.50	12.18	3.41	3.42	4.71	2.78	20.66
1666	rpffio	2	64.10	8.00	2.40	10.15	1.97	2.98	4.47	2.34	20.69
1786	rpfrpf	2	51.65	8.00	1.80	11.78	3.17	3.44	4.49	2.75	26.25
1826	rpfrpf	2	47.55	8.00	2.00	11.19	2.68	3.13	4.65	2.55	23.94
1866	rpffio	1	42.00	8.00	1.90	11.73	2.76	3.43	4.83	2.80	24.83
1876	rpffio	2	38.10	8.00	1.90	11.82	2.79	3.40	4.84	2.82	23.89
2096	rpffio	2	46.20	8.00	2.80	11.28	2.41	3.29	4.75	2.63	26.23
2106	rpffio	2	50.20	8.00	2.60	11.85	3.10	3.14	4.89	2.59	22.34
2126	fiofio	1	45.50	8.00	1.70	13.52	4.48	3.46	4.85	2.94	24.93
