# Supplementary material for: Effects of prenatal fish oil supplementation on the development and performance of female kids after weaning
Source: PLoS One. 2024 Sep 11;19(9):e0310220. doi: 10.1371/journal.pone.0310220 (PMC11389935; doi:10.1371/journal.pone.0310220)
Supplement: S7 Appendix — (PDF) [file pone.0310220.s008.pdf]

atagno	dbbw	kidtagno	trt	sex	pregtype	birthweigh	time	bw
1876	38.10	3128	rpffio	Female	2	2.20	14.00	2.75
1876	38.10	3138	rpffio	Female	2	1.90	14.00	2.10
1866	42.00	2108	rpffio	Female	1	2.40	14.00	6.95
1396	66.00	3178	rpffio	Male	2	3.40	14.00	.
1396	66.00	3189	rpffio	Female	2	2.80	14.00	.
1016	44.70	2708	rpffio	Male	1	3.35	14.00	8.00
2096	46.20	1798	rpffio	Female	2	3.00	14.00	8.40
2096	46.20	1808	rpffio	Female	2	3.05	14.00	8.10
1666	64.10	2468	rpffio	Female	2	2.98	14.00	7.40
1666	64.10	2478	rpffio	Female	2	2.89	14.00	6.50
2106	50.20	2668	rpffio	Female	2	3.77	14.00	6.50
2106	50.20	2678	rpffio	Male	2	3.48	14.00	7.10
1656	44.10	2048	rpffio	Female	1	3.50	14.00	8.20
1026	51.00	2158	rpffio	Male	2	2.80	14.00	7.30
1026	51.00	2168	rpffio	Female	2	2.50	14.00	7.00
1066	45.35	2028	rpffio	Male	2	3.25	14.00	7.70
1066	45.35	2038	rpffio	Female	2	3.20	14.00	6.45
1056	46.75	1988	rpffio	Male	2	2.73	14.00	7.30
1056	46.75	1998	rpffio	Female	2	2.33	14.00	6.45
1096	63.20	2188	rpffio	Female	2	2.35	14.00	6.00
1096	63.20	2198	rpffio	Female	2	2.65	14.00	6.70
1106	55.25	2388	rpffio	Male	2	3.39	14.00	6.25
1106	55.25	2398	rpffio	Male	2	3.10	14.00	6.85
1546	53.45	2358	rpffio	Male	2	3.65	14.00	8.50
1316	45.35	2698	fiofio	Female	2	2.56	14.00	4.00
1456	46.60	2688	fiofio	Male	2	2.85	14.00	6.20
1316	45.35	3108	fiofio	Female	1	3.40	14.00	6.45
1366	46.25	2748	fiofio	Male	2	2.95	14.00	5.20
1366	46.25	2758	fiofio	Male	2	3.00	14.00	6.15
1356	45.15	2848	fiofio	Female	2	3.50	14.00	5.45
1356	45.15	2858	fiofio	Male	2	3.25	14.00	5.40
1486	43.60	2948	fiofio	Female	2	2.38	14.00	4.50
1486	43.60	2958	fiofio	Female	2	2.63	14.00	5.40
9260	57.70	2718	fiofio	Female	1	3.63	14.00	6.10
2126	45.50	2568	fiofio	Female	1	3.45	14.00	6.50
1446	48.70	3168	fiofio	Female	1	3.80	14.00	.
1526	41.15	2118	fiorpf	Male	2	2.30	14.00	6.45
1476	51.90	2598	fiorpf	Female	1	3.40	14.00	7.60
1896	46.35	2768	fiorpf	Male	1	4.35	14.00	6.20
1166	52.00	2098	fiorpf	Male	1	3.90	14.00	10.10
1376	58.70	2408	fiorpf	Male	2	3.85	14.00	6.40
1376	58.70	2418	fiorpf	Female	2	3.52	14.00	7.10
1126	50.80	2578	rpfrpf	Male	2	2.95	14.00	6.20
1126	50.80	2588	rpfrpf	Female	2	2.80	14.00	6.40
1416	47.70	2648	rpfrpf	Male	2	3.34	14.00	6.60
1416	47.70	2658	rpfrpf	Female	2	3.15	14.00	5.80
1426	44.40	2938	rpfrpf	Male	1	3.70	14.00	6.90
1786	51.65	2778	rpfrpf	Male	2	3.25	14.00	6.10
1786	51.65	2788	rpfrpf	Female	2	3.12	14.00	5.50

1826	47.55	2368	rpfrpf	Male	2	3.55	14.00	6.40
1826	47.55	2378	rpfrpf	Male	2	3.25	14.00	6.10
1616	53.95	2488	rpfrpf	Female	2	2.85	14.00	6.30
1616	53.95	2498	rpfrpf	Male	2	3.10	14.00	7.10
1506	52.35	2628	rpfrpf	Male	2	3.25	14.00	5.30
1506	52.35	2638	rpfrpf	Male	2	3.16	14.00	5.90
1086	47.25	2208	rpfrpf	Female	2	3.00	14.00	6.50
1086	47.25	2218	rpfrpf	Female	2	2.95	14.00	6.00
1876	38.10	3128	rpffio	Female	2	2.20	28.00	4.20
1876	38.10	3138	rpffio	Female	2	1.90	28.00	2.95
1866	42.00	2108	rpffio	Female	1	2.40	28.00	9.25
1396	66.00	3178	rpffio	Male	2	3.40	28.00	3.70
1396	66.00	3189	rpffio	Female	2	2.80	28.00	3.20
1016	44.70	2708	rpffio	Male	1	3.35	28.00	8.90
2096	46.20	1798	rpffio	Female	2	3.00	28.00	10.40
2096	46.20	1808	rpffio	Female	2	3.05	28.00	10.40
1666	64.10	2468	rpffio	Female	2	2.98	28.00	9.50
1666	64.10	2478	rpffio	Female	2	2.89	28.00	7.75
2106	50.20	2668	rpffio	Female	2	3.77	28.00	8.30
2106	50.20	2678	rpffio	Male	2	3.48	28.00	9.30
1656	44.10	2048	rpffio	Female	1	3.50	28.00	11.00
1026	51.00	2158	rpffio	Male	2	2.80	28.00	10.10
1026	51.00	2168	rpffio	Female	2	2.50	28.00	8.95
1066	45.35	2028	rpffio	Male	2	3.25	28.00	9.30
1066	45.35	2038	rpffio	Female	2	3.20	28.00	9.00
1056	46.75	1988	rpffio	Male	2	2.73	28.00	9.50
1056	46.75	1998	rpffio	Female	2	2.33	28.00	6.40
1096	63.20	2188	rpffio	Female	2	2.35	28.00	6.50
1096	63.20	2198	rpffio	Female	2	2.65	28.00	8.75
1106	55.25	2388	rpffio	Male	2	3.39	28.00	8.10
1106	55.25	2398	rpffio	Male	2	3.10	28.00	6.95
1546	53.45	2358	rpffio	Male	2	3.65	28.00	11.45
1316	45.35	2698	fiofio	Female	2	2.56	28.00	5.30
1456	46.60	2688	fiofio	Male	2	2.85	28.00	7.70
1316	45.35	3108	fiofio	Female	1	3.40	28.00	8.00
1366	46.25	2748	fiofio	Male	2	2.95	28.00	6.30
1366	46.25	2758	fiofio	Male	2	3.00	28.00	8.00
1356	45.15	2848	fiofio	Female	2	3.50	28.00	7.50
1356	45.15	2858	fiofio	Male	2	3.25	28.00	4.10
1486	43.60	2948	fiofio	Female	2	2.38	28.00	6.35
1486	43.60	2958	fiofio	Female	2	2.63	28.00	7.30
9260	57.70	2718	fiofio	Female	1	3.63	28.00	8.10
2126	45.50	2568	fiofio	Female	1	3.45	28.00	6.00
1446	48.70	3168	fiofio	Female	1	3.80	28.00	4.50
1526	41.15	2118	fiorpf	Male	2	2.30	28.00	9.50
1476	51.90	2598	fiorpf	Female	1	3.40	28.00	7.65
1896	46.35	2768	fiorpf	Male	1	4.35	28.00	8.45
1166	52.00	2098	fiorpf	Male	1	3.90	28.00	12.45
1376	58.70	2408	fiorpf	Male	2	3.85	28.00	6.35
1376	58.70	2418	fiorpf	Female	2	3.52	28.00	8.90

1126	50.80	2578	rpfrpf	Male	2	2.95	28.00	8.20
1126	50.80	2588	rpfrpf	Female	2	2.80	28.00	8.00
1416	47.70	2648	rpfrpf	Male	2	3.34	28.00	8.45
1416	47.70	2658	rpfrpf	Female	2	3.15	28.00	7.70
1426	44.40	2938	rpfrpf	Male	1	3.70	28.00	8.70
1786	51.65	2778	rpfrpf	Male	2	3.25	28.00	5.60
1786	51.65	2788	rpfrpf	Female	2	3.12	28.00	5.90
1826	47.55	2368	rpfrpf	Male	2	3.55	28.00	7.50
1826	47.55	2378	rpfrpf	Male	2	3.25	28.00	8.25
1616	53.95	2488	rpfrpf	Female	2	2.85	28.00	6.75
1616	53.95	2498	rpfrpf	Male	2	3.10	28.00	9.10
1506	52.35	2628	rpfrpf	Male	2	3.25	28.00	4.60
1506	52.35	2638	rpfrpf	Male	2	3.16	28.00	7.50
1086	47.25	2208	rpfrpf	Female	2	3.00	28.00	6.80
1086	47.25	2218	rpfrpf	Female	2	2.95	28.00	8.30
1876	38.10	3128	rpffio	Female	2	2.20	42.00	4.50
1876	38.10	3138	rpffio	Female	2	1.90	42.00	4.50
1866	42.00	2108	rpffio	Female	1	2.40	42.00	9.80
1396	66.00	3178	rpffio	Male	2	3.40	42.00	4.30
1396	66.00	3189	rpffio	Female	2	2.80	42.00	4.10
1016	44.70	2708	rpffio	Male	1	3.35	42.00	11.40
2096	46.20	1798	rpffio	Female	2	3.00	42.00	11.40
2096	46.20	1808	rpffio	Female	2	3.05	42.00	11.90
1666	64.10	2468	rpffio	Female	2	2.98	42.00	11.30
1666	64.10	2478	rpffio	Female	2	2.89	42.00	10.20
2106	50.20	2668	rpffio	Female	2	3.77	42.00	10.00
2106	50.20	2678	rpffio	Male	2	3.48	42.00	10.30
1656	44.10	2048	rpffio	Female	1	3.50	42.00	13.20
1026	51.00	2158	rpffio	Male	2	2.80	42.00	11.90
1026	51.00	2168	rpffio	Female	2	2.50	42.00	10.60
1066	45.35	2028	rpffio	Male	2	3.25	42.00	10.30
1066	45.35	2038	rpffio	Female	2	3.20	42.00	11.00
1056	46.75	1988	rpffio	Male	2	2.73	42.00	11.20
1056	46.75	1998	rpffio	Female	2	2.33	42.00	9.10
1096	63.20	2188	rpffio	Female	2	2.35	42.00	9.30
1096	63.20	2198	rpffio	Female	2	2.65	42.00	9.80
1106	55.25	2388	rpffio	Male	2	3.39	42.00	9.80
1106	55.25	2398	rpffio	Male	2	3.10	42.00	10.90
1546	53.45	2358	rpffio	Male	2	3.65	42.00	14.30
1316	45.35	2698	fiofio	Female	2	2.56	42.00	6.10
1456	46.60	2688	fiofio	Male	2	2.85	42.00	8.30
1316	45.35	3108	fiofio	Female	1	3.40	42.00	9.50
1366	46.25	2748	fiofio	Male	2	2.95	42.00	7.80
1366	46.25	2758	fiofio	Male	2	3.00	42.00	9.80
1356	45.15	2848	fiofio	Female	2	3.50	42.00	8.80
1356	45.15	2858	fiofio	Male	2	3.25	42.00	7.50
1486	43.60	2948	fiofio	Female	2	2.38	42.00	7.30
1486	43.60	2958	fiofio	Female	2	2.63	42.00	9.20
9260	57.70	2718	fiofio	Female	1	3.63	42.00	9.70
2126	45.50	2568	fiofio	Female	1	3.45	42.00	8.85

1446	48.70	3168	fiofio	Female	1	3.80	42.00	5.50
1526	41.15	2118	fiorpf	Male	2	2.30	42.00	11.20
1476	51.90	2598	fiorpf	Female	1	3.40	42.00	11.40
1896	46.35	2768	fiorpf	Male	1	4.35	42.00	9.00
1166	52.00	2098	fiorpf	Male	1	3.90	42.00	14.60
1376	58.70	2408	fiorpf	Male	2	3.85	42.00	10.30
1376	58.70	2418	fiorpf	Female	2	3.52	42.00	9.70
1126	50.80	2578	rpfrpf	Male	2	2.95	42.00	9.80
1126	50.80	2588	rpfrpf	Female	2	2.80	42.00	9.70
1416	47.70	2648	rpfrpf	Male	2	3.34	42.00	8.70
1416	47.70	2658	rpfrpf	Female	2	3.15	42.00	9.10
1426	44.40	2938	rpfrpf	Male	1	3.70	42.00	9.70
1786	51.65	2778	rpfrpf	Male	2	3.25	42.00	9.30
1786	51.65	2788	rpfrpf	Female	2	3.12	42.00	7.50
1826	47.55	2368	rpfrpf	Male	2	3.55	42.00	7.50
1826	47.55	2378	rpfrpf	Male	2	3.25	42.00	9.20
1616	53.95	2488	rpfrpf	Female	2	2.85	42.00	9.80
1616	53.95	2498	rpfrpf	Male	2	3.10	42.00	10.30
1506	52.35	2628	rpfrpf	Male	2	3.25	42.00	8.30
1506	52.35	2638	rpfrpf	Male	2	3.16	42.00	9.10
1086	47.25	2208	rpfrpf	Female	2	3.00	42.00	9.50
1086	47.25	2218	rpfrpf	Female	2	2.95	42.00	8.30
1876	38.10	3128	rpffio	Female	2	2.20	56.00	5.50
1876	38.10	3138	rpffio	Female	2	1.90	56.00	4.80
1866	42.00	2108	rpffio	Female	1	2.40	56.00	9.20
1396	66.00	3178	rpffio	Male	2	3.40	56.00	5.00
1396	66.00	3189	rpffio	Female	2	2.80	56.00	5.20
1016	44.70	2708	rpffio	Male	1	3.35	56.00	11.70
2096	46.20	1798	rpffio	Female	2	3.00	56.00	13.20
2096	46.20	1808	rpffio	Female	2	3.05	56.00	14.30
1666	64.10	2468	rpffio	Female	2	2.98	56.00	12.50
1666	64.10	2478	rpffio	Female	2	2.89	56.00	10.70
2106	50.20	2668	rpffio	Female	2	3.77	56.00	10.30
2106	50.20	2678	rpffio	Male	2	3.48	56.00	10.30
1656	44.10	2048	rpffio	Female	1	3.50	56.00	13.50
1026	51.00	2158	rpffio	Male	2	2.80	56.00	14.30
1026	51.00	2168	rpffio	Female	2	2.50	56.00	11.90
1066	45.35	2028	rpffio	Male	2	3.25	56.00	12.30
1066	45.35	2038	rpffio	Female	2	3.20	56.00	13.00
1056	46.75	1988	rpffio	Male	2	2.73	56.00	13.00
1056	46.75	1998	rpffio	Female	2	2.33	56.00	11.80
1096	63.20	2188	rpffio	Female	2	2.35	56.00	10.30
1096	63.20	2198	rpffio	Female	2	2.65	56.00	11.20
1106	55.25	2388	rpffio	Male	2	3.39	56.00	11.30
1106	55.25	2398	rpffio	Male	2	3.10	56.00	12.00
1546	53.45	2358	rpffio	Male	2	3.65	56.00	14.70
1316	45.35	2698	fiofio	Female	2	2.56	56.00	7.70
1456	46.60	2688	fiofio	Male	2	2.85	56.00	8.00
1316	45.35	3108	fiofio	Female	1	3.40	56.00	10.20
1366	46.25	2748	fiofio	Male	2	2.95	56.00	9.70

1366	46.25	2758	fiofio	Male	2	3.00	56.00	10.30
1356	45.15	2848	fiofio	Female	2	3.50	56.00	10.20
1356	45.15	2858	fiofio	Male	2	3.25	56.00	8.60
1486	43.60	2948	fiofio	Female	2	2.38	56.00	8.80
1486	43.60	2958	fiofio	Female	2	2.63	56.00	8.60
9260	57.70	2718	fiofio	Female	1	3.63	56.00	10.50
2126	45.50	2568	fiofio	Female	1	3.45	56.00	10.30
1446	48.70	3168	fiofio	Female	1	3.80	56.00	6.00
1526	41.15	2118	fiorpf	Male	2	2.30	56.00	9.60
1476	51.90	2598	fiorpf	Female	1	3.40	56.00	13.40
1896	46.35	2768	fiorpf	Male	1	4.35	56.00	9.50
1166	52.00	2098	fiorpf	Male	1	3.90	56.00	16.30
1376	58.70	2408	fiorpf	Male	2	3.85	56.00	11.00
1376	58.70	2418	fiorpf	Female	2	3.52	56.00	10.70
1126	50.80	2578	rpfrpf	Male	2	2.95	56.00	9.50
1126	50.80	2588	rpfrpf	Female	2	2.80	56.00	9.00
1416	47.70	2648	rpfrpf	Male	2	3.34	56.00	10.60
1416	47.70	2658	rpfrpf	Female	2	3.15	56.00	9.75
1426	44.40	2938	rpfrpf	Male	1	3.70	56.00	10.70
1786	51.65	2778	rpfrpf	Male	2	3.25	56.00	10.80
1786	51.65	2788	rpfrpf	Female	2	3.12	56.00	8.40
1826	47.55	2368	rpfrpf	Male	2	3.55	56.00	7.30
1826	47.55	2378	rpfrpf	Male	2	3.25	56.00	10.70
1616	53.95	2488	rpfrpf	Female	2	2.85	56.00	10.60
1616	53.95	2498	rpfrpf	Male	2	3.10	56.00	11.20
1506	52.35	2628	rpfrpf	Male	2	3.25	56.00	9.10
1506	52.35	2638	rpfrpf	Male	2	3.16	56.00	9.20
1086	47.25	2208	rpfrpf	Female	2	3.00	56.00	10.10
1086	47.25	2218	rpfrpf	Female	2	2.95	56.00	7.70
